# Investigating Core Signaling Pathways of Hepatitis B Virus Pathogenesis for Biomarkers Identification and Drug Discovery via Systems Biology and Deep Learning Method

**DOI:** 10.3390/biomedicines8090320

**Published:** 2020-08-31

**Authors:** Shen Chang, Lily Hui-Ching Wang, Bor-Sen Chen

**Affiliations:** 1 Laboratory of Automatic Control, Signal Processing and Systems Biology, Department of Electrical Engineering, National Tsing Hua University, Hsinchu 30013, Taiwan; s107061595@m107.nthu.edu.tw; 2 Institute of Molecular and Cellular Biology, National Tsing Hua University, Hsinchu 30013, Taiwan; lilywang@life.nthu.edu.tw

**Keywords:** hepatitis B virus infection, pathogenesis, host/pathogen interspecies genetic and epigenetic network (HPI-GEN), systems medicine discovery, drug-target interaction (DTI) model, deep learning, multiple-molecule drug

## Abstract

Hepatitis B Virus (HBV) infection is a major cause of morbidity and mortality worldwide. However, poor understanding of its pathogenesis often gives rise to intractable immune escape and prognosis recurrence. Thus, a valid systematic approach based on big data mining and genome-wide RNA-seq data is imperative to further investigate the pathogenetic mechanism and identify biomarkers for drug design. In this study, systems biology method was applied to trim false positives from the host/pathogen genetic and epigenetic interaction network (HPI-GEN) under HBV infection by two-side RNA-seq data. Then, via the principal network projection (PNP) approach and the annotation of KEGG (Kyoto Encyclopedia of Genes and Genomes) pathways, significant biomarkers related to cellular dysfunctions were identified from the core cross-talk signaling pathways as drug targets. Further, based on the pre-trained deep learning-based drug-target interaction (DTI) model and the validated pharmacological properties from databases, i.e., drug regulation ability, toxicity, and sensitivity, a combination of promising multi-target drugs was designed as a multiple-molecule drug to create more possibility for the treatment of HBV infection. Therefore, with the proposed systems medicine discovery and repositioning procedure, we not only shed light on the etiologic mechanism during HBV infection but also efficiently provided a potential drug combination for therapeutic treatment of Hepatitis B.

## 1. Introduction

Hepatitis B Virus (HBV) infection remains a prevalent health challenge worldwide, and is greatly associated with substantial morbidity and mortality due to serious liver diseases, such as hepatitis, fibrosis, cirrhosis, and even hepatocellular carcinoma [1]. According to the latest report of World Health Organization (WHO) [2], HBV affected an estimated 257 million people and causing 600,000 deaths per year. While current therapies seek to control the progression of the disease, life-long treatment and surveillance are still needed because resistance develops during treatment and reactivation often occurs after medication discontinuation [3,4]. In recent years, dedication to new drug design and immunotherapy development has been made by researchers to hopefully silence HBV in infected hepatocytes [5,6]. However, the host/pathogen cross-talk mechanism contributing to the development and persistence of HBV infection requires further investigation, and its current therapies are still inadequate. Thus, new treatment options are needed to achieve a better therapy for HBV-infected patients.

Even so, de novo drug discovery is particularly an expensive, time-consuming, and high-risk process, let alone exploring a drug to eradicate HBV from patients, which is generally believed arduous [4,7,8]. Conventionally, the pipelines of drug development from laboratory to market take nearly average 13 to 15 years and cost approximately two to three billion U.S. dollars, in which about 10% of all drugs that start from pre-clinical trials ever make it to actual human testing [9,10]. Fortunately, as the advancement of biological science drives the progress of high-throughput functional genomics and proteomics technology, novel experimental and bioinformatics methods have not only accelerated the deciphering of protein interactions, but also given rise to the construction of networks for signaling pathways and protein complex identification in specific diseases [11]. Meanwhile, based on the manifold data types from wet-lab experiments, several computational approaches have been suggested to predict biological networks for expediting the modeling of functional pathways so as to exemplify the molecular mechanisms of cellular processes. Researchers are thereby being empowered to profoundly explore the complicated and mysterious mechanisms of biological systems for better investigating the potential drug targets [12]. On the other hand, with the development of molecular biology, drug repurposing, also known as drug repositioning, was proposed to reduce the time spent and the capital expenditure. The process of drug repositioning is to find new use outside the scope of the original medical indication for existing drugs [13]. Its prospect for new clinical indications is promising, since compared with other drug development strategies, a repositioned drug could be instantly put into preclinical testing and clinical trials for its safety and reliability proven in humans.

Nonetheless, the commonly used correlation networks in bioinformatics applications are often based on oversimplified algorithms. As inherent difficulties appear in assessing the impact of microRNA (miRNA), long non-coding RNA (lncRNA), epigenetic modification, microenvironment factors, etc., hardly can these networks mirror the complicated mechanisms of factual biological systems. In addition, while wide doses of the repositioned drugs can be tested using in vitro and in vivo experiments, it takes great time and effort. Therefore, to discover potential drugs for the treatment of HBV-infected patients, the development of a systematic procedure in computational framework based on potent models and existing data is indispensable.

In this study, we employed the systems biology technique to identify the significant biomarkers from the host/pathogen interspecies genetic and epigenetic interaction network (HPI-GEN) through two-side RNA-seq data during HBV infection. Subsequently, with the analysis of these biomarkers by the proposed drug discovery and repositioning procedure, potential combinations of multi-targets drugs were recommended to the clinical trials of Hepatitis B therapy. By and large, the process can be subdivided into a few steps: (1) Construct candidate HPI-GEN composed of host/pathogen interspecies protein-protein interaction network (HPI-PPIN) and host/pathogen interspecies gene regulation network (HPI-GRN) by big data mining. (2) Prune the false positives in candidate HPI-GEN by system identification analysis with the assistance of two-side profile RNA-seq data and Akaike Information Criterion (AIC) to identify the real HPI-GEN between host and pathogen. (3) Extract the core HPI-GEN from real HPI-GEN via principal network projection (PNP) approach. (4) Investigate the core signaling pathways related to manifold cellular function (especially host inflammatory and immune response, pathological anti-apoptosis, and cell survival) in the core HPI-GEN by the annotation of KEGG (Kyoto Encyclopedia of Genes and Genomes) pathways to gain insights into the etiologic mechanism of HBV infection. (5) Select the significant biomarkers related to pathological cellular dysfunctions. (6) Predict the candidate drugs from available drugs for the selected significant biomarkers by the pre-trained deep learning-based drug-target interaction (DTI) model. (7) Consider general criteria for drug design specifications including regulation ability, toxicity, and sensitivity to further sift out the promising drugs. (8) Combine these agreeable drugs as a potential multiple-molecule drug for possible therapeutic treatment of Hepatitis B. It is worthwhile to note that instead of a single drug, a combination of promising drugs was proposed for the treatment to slow or even prevent inherent or adaptive drug resistance through the simultaneous blockade of multiple targets or pathways [14].

Nowadays, researches have been dedicated to drug discovery and design, while few of them propose an integral end-to-end systematic procedure from identifying significant biomarkers based on pathogenic mechanism to recommending promising drugs for one disease. Here, we dig into the pathogenic characters of viral proteins played on the pathogenesis to select the potential drug targets from two-side RNA-seq data and reinterpret the role of available drugs based on a pre-trained deep learning-based DTI model through big drug-target interaction data, which gives an alternative way for systems drug discovery and repositioning to overcome HBV infection.

## 2. Materials and Methods 

### 2.1. The Construction of the HPI-GEN in HepaRG Cell Line during HBV Infection and the Application of Deep Learning-Based DTI Model for New Drug Design: An Overview

To gain new insight into the molecular mechanism of HBV infection, candidate cross-talk HPI-GEN, which consists of candidate intraspecies, candidate interspecies PPIN between host and pathogen, and candidate HPI-GRN encompassing regulatory networks between host gene, pathogen gene, host miRNA and host lncRNA, was constructed by big data mining and preprocessing of host/pathogen gene/miRNA/lncRNA expression from online database. 

Given that the information for interspecies PPIN construction is insufficient, in addition to interactions attained from bio-databases, data amplification technique through nature-linguistic programming (NLP) and web scrapping was introduced to expand the dataset. Then, for identifying real HPI-GEN, a systematic identification approach was exploited based on the genome-wide RNA-seq data of HepaRG cells and Hepatitis B Virus under HBV infection. In addition, to deal with the highly complicate real HPI-GEN, we employed PNP method to extract core network structure consisted of the top 3000 nodes with highest energy. Such effective approach benefits us to investigate the core signaling pathways for establishing the crucial molecular pathogenic mechanism to identify significant biomarkers that contribute to the progression of HBV infection. Finally, following the drug discovery procedure, we built the deep learning-based DTI model to identify the potential multi-target drugs to target their significant biomarkers for therapeutic treatment of HBV infection.

### 2.2. Big Data Mining and Data Preprocessing of RNA-seq Data for Human and Pathogen

In this study, two-side sequencing-based dataset was obtained from the Gene Expression Omnibus (GEO) at the National Center for Biotechnology Information (NCBI). We obtained the data recorded by Mueller et al. [15], containing human and virus genomes with the time-course through GEO series with accession number GSE101575 [15]. The raw data of the dataset, which contains 27,267 human genes and 4 HBV genes, involves the gene expression profiles of human HepaRG cells from three patients during HBV infection at 6, 9, 12, and 24 h post reactivation with drug (RG7834) treatment and without. Only the data without medication administration was deployed to the ensuing system identification process. 

To unravel the cellular mechanism during HBV infection, core signaling pathways identified from HPI-GEN are requisite. The candidate HPI-GEN, involving many experimental data and computational predictions from numerous databases, consists of multiple networks. Among them, the candidate human PPIs were obtained from the Biological General Repository for Interaction Datasets database (BioGRID) [16], DIP [17], BIND [18], IntAct [19], and MINT [20]; the candidate human miRNA and lncRNA regulations were individually obtained from Target Scan Human database [21], and CircuitDB [22]; the candidate regulations between human transcription factors and their targets were according to the Integrated Transcription Factor Platform database (ITFP) [23], the Human Transcriptional Regulation Interactions database (HTRIdb) [24], and the Transcription Factor database (TRANSFAC) [25]; the candidate host-pathogen interspecies PPIs were obtained from VirusMentha [26], IMEx [27], PSICQUIC [28], the PPIN constructed by Zhong et al. [29], and based on the automatic pipeline method of NLP; the candidate host-pathogen interspecies regulations were collected from VIRmiRNA [30], ViRBase [31], miRecords [32], starBase v2.0 [33], miRTarBase [34], and based on the automatic pipeline method of NLP. 

### 2.3. Text Mining of Human and Pathogen Protein Interactions

To reinforce the comprehension of the interaction relationships between host and pathogen, BioBERT (Bidirectional Encoder Representations from Transformer for Biomedical Text Mining) [35,36], an up-to-date BioNER (Biomedical Named Entity Recognition) approach is employed on the PubMed database to analyze binary interactions between host and pathogen. BioNER is a computerized procedure of identifying biomedical named entities such as genes, protein complexes, diseases, and chemicals in texts referring to information retrieval and data extraction. Amongst all the BioNER application, BioBERT, which has achieved state-of-the-art performance in terms of precision, is efficaciously utilized in biomedical text mining applications to address data.

In brief, we scraped and collected 26,732 existing PubMed articles associated with Hepatitis B Virus and Homo sapiens within 20 years and transformed it into XML format. Nonetheless, on account of Pubmed’s property, the full texts from most articles in PMC aren’t publicly accessible so that looking forward to automatically download integrated content is unrealistic. Consequently, we additionally crawled 2831 articles from PLOS ONE to strengthen our network complexity. Then, BioBERT model was exerted to discern known entities and identify the types of overlapping entities among each sentence separated from the relevant content with periods in the corpus. 

In order to facilitate analysis and reconfirmation, we also gathered the synonym names of both host and pathogen proteins (compiled from BioThesaurus database [37]) to prevent omission of vital interactions owing to protein names mismatch. Afterwards, in each sentence, we extracted all present proteins and identified the correlations by determining the human proteins under the circumstance that Hepatitis B Virus proteins had existed. To abate computational complexity, in conformity with the subsequent system identification processing requirement, the verb recognition and identification were left out of account and binary interactions were built. For instance, providing that “HBx” and “BCL2” were simultaneously identified in the same sentence, we then set the corresponding value to 1 and so forth, vice versa to 0. 

Furthermore, to verify that authentic interaction relationships derived from the statistical analysis of sentences, we scrutinized entire extracted entities and completed the construction of ultimate host and pathogen interaction-relationships matrix.

### 2.4. Dynamic Models of Candidate HPI-GEN for Human Cells and HBV During the Infection

Candidate HPI-GEN, constructed via big data mining techniques, is the epitome of an intricate network comprised of protein interactions and gene regulations between host and pathogen during HBV infection. As inevitable false-positive information extraction might incur system distraction from genuine consequence and discrepancy during the pathway analysis process, dynamic models were built to carry out systematic identification approach by two-side time-profile HBV infection RNA-seq data to prune false-positives in candidate HPI-GEN.

Protein abundances mirror a dynamic balance among a series of linked processes spanning the transcription, processing, and degradation of mRNAs to the translation, modification, and breakdown of the proteins themselves necessary for cellular protein production and maintenance [38]. As a consequence, for the protein interactive model of PPI pre-network in candidate HPI-GEN, the dynamic interaction model of the *i*th host protein can be described by the following equation:

(1) $$\begin{array}{l} p_{i}^{H}\left(t+1\right)=p_{i}^{H}\left(t\right)+\sum_{n=1}^{N_{i}}a_{in}^{H}p_{i}^{H}\left(t\right)p_{n}^{H}\left(t\right)+\sum_{v=1}^{V_{i}}b_{iv}^{H}p_{i}^{H}\left(t\right)p_{v}^{P}\left(t\right)\\ +\alpha_{i}^{H}g_{i}^{H}\left(t\right)-\gamma_{i}^{H}p_{i}^{H}\left(t\right)+\beta_{i}^{H}+v_{i}^{H}\left(t\right),\;\mathrm{for}\;i=1,2,\ldots ,I,\;\alpha_{i}^{H}\geq 0\;\mathrm{and}\;-\gamma_{i}^{H}\leq 0 \end{array}$$

where $p_{i}^{H}\left(t\right),\;p_{n}^{H}\left(t\right),\;g_{i}^{H}\left(t\right)$ and $p_{v}^{P}\left(t\right)$ indicate the expression levels of the *i*th host protein, the *n*th host protein, the *i*th host gene, and the *v*th pathogen protein at time *t*, respectively; $a_{in}^{H}$ and $b_{iv}^{H}$ denote the interactive ability between the *i*th host protein and *n*th host protein and between the *i*th host protein and *v*th pathogen protein, respectively; $N_{i}\;\mathrm{and}\;V_{i}$ signify the number of host proteins and pathogen proteins interacting with the *i*th host protein; and I typifies the total number of the human proteins in candidate PPIN. $\alpha_{i}^{H},\;-\gamma_{i}^{H}$ and $\beta_{i}^{H}$ indicate the translation rate from the corresponding mRNA, the degradation rate, and the basal expression level of the *i*th host protein, respectively; the basal level denotes the interactions with unknown factors, such as acetylation, methylation, and ubiquitination; and $v_{i}^{H}\left(t\right)$ represents the stochastic noise of the *i*th host protein at time *t*. Note that the intraspecies and interspecies biological mechanism of human proteins in candidate PPIN are represented in Equation (1) as the form of $\sum_{n=1}^{N_{i}}a_{in}^{H}p_{i}^{H}\left(t\right)p_{n}^{H}\left(t\right)\;$ and $\sum_{v=1}^{V_{i}}b_{iv}^{H}p_{i}^{H}\left(t\right)p_{v}^{P}\left(t\right)$, respectively. 

Now that the interactions between the host and pathogen proteins are mutual and their biological characteristics involved in PPIs are similar, the dynamic PPIN of the qth pathogen protein in candidate HPI-GEN can be described by the analogous equation as follows:

(2) $$\begin{array}{l} p_{q}^{P}\left(t+1\right)=p_{q}^{P}\left(t\right)+\sum_{n=1}^{N_{q}}a_{qn}^{P}p_{q}^{P}\left(t\right)p_{n}^{H}\left(t\right)+\sum_{v=1}^{V_{q}}b_{qv}^{P}p_{q}^{P}\left(t\right)p_{v}^{P}\left(t\right)\\ +\alpha_{q}^{P}g_{q}^{P}\left(t\right)-\gamma_{q}^{P}p_{q}^{P}\left(t\right)+\beta_{q}^{P}+v_{q}^{P}\left(t\right),\;\mathrm{for}\;q=1,2,\ldots ,Q,\;\alpha_{q}^{P}\geq 0\;\mathrm{and}\;-\gamma_{q}^{P}\leq 0 \end{array}$$

where $p_{q}^{P}\left(t\right),\;p_{n}^{H}\left(t\right),\;g_{q}^{P}\left(t\right)$ and $p_{v}^{P}\left(t\right)$ indicate the expression levels of the qth pathogen protein, the *n*th host protein, the qth pathogen gene, and the *v*th pathogen protein at time *t*, respectively; $a_{qn}^{P}$ and $b_{qv}^{P}$ denote the interactive ability between the qth pathogen protein and *n*th host protein and between the qth pathogen protein and *v*th pathogen protein, respectively; $N_{q}$ and $V_{q}$ signify the number of host proteins and pathogen proteins interacting with the qth pathogen protein; and Q typifies the total number of the pathogen proteins in candidate PPIN. $\alpha_{q}^{P},\;-\gamma_{q}^{P}$ and $\beta_{q}^{P}$ indicate the translation rate from the corresponding mRNA, the degradation rate, and the basal expression level of the qth pathogen protein, respectively; the basal level $\beta_{q}^{P}$ denotes the interactions with unknown factors such as acetylation and ubiquitination; and $v_{q}^{P}\left(t\right)$ represents the stochastic noise of the qth pathogen protein at time *t*. Similarly, the intraspecies and interspecies biological mechanism of pathogen proteins in candidate PPIN are represented in Equation (2) as the form of $\sum_{n=1}^{N_{q}}a_{qn}^{P}p_{q}^{P}\left(t\right)p_{n}^{P}\left(t\right)\;$ and $\sum_{v=1}^{V_{q}}b_{qv}^{P}p_{q}^{P}\left(t\right)p_{v}^{H}\left(t\right)$, respectively. 

For the GRN of host genes in the candidate HPI-GEN, the dynamic regulatory model of the *j*th host gene can be described as follows:

(3) $$\begin{array}{l} g_{j}^{H}\left(t+1\right)=g_{j}^{H}\left(t\right)+\sum_{\tau =1}^{T_{j}}c_{j\tau }^{H}p_{\tau }^{H}\left(t\right)+\sum_{\ell =1}^{L_{j}}d_{j\ell }^{H}l_{\ell }^{H}\left(t\right)-\sum_{\mu =1}^{M_{j}}e_{j\mu }^{H}g_{j}^{H}\left(t\right)m_{\mu }^{H}\left(t\right)\\ -\lambda_{j}^{H}g_{j}^{H}\left(t\right)+\delta_{j}^{H}+\varpi_{j}^{H}\left(t\right),\;\mathrm{for}\;j=1,2,\ldots ,J,\;-e_{j\mu }^{H}\leq 0\;\mathrm{and}-\lambda_{j}^{H}\leq 0 \end{array}$$

where $g_{j}^{H}\left(t\right),\;p_{\tau }^{H}\left(t\right),\;m_{\mu }^{H}\left(t\right)$ and $l_{\ell }^{H}\left(t\right)$ indicate the expression levels of the *j*th host gene, the τth host TF, the μth host microRNA, and the ℓth host lncRNA at time *t*, respectively; $c_{j\tau }^{H},\;-e_{j\mu }^{H}$ and $d_{j\ell }^{H}$ represent the regulation ability of the τth host TF, the μth host microRNA, and the ℓth host lncRNA on the *j*th host gene, respectively; $T_{j},\;M_{j}$ and $L_{j}$ denote the number of host TFs, host miRNAs, and host lncRNAs, respectively, which regulate the expression level of the *j*th host gene; J indicates the total amount of genes in GRN; $-\lambda_{j}^{H}$ and $\delta_{j}^{H}$ indicate the degradation rate and the expression basal level of the *j*th host gene, respectively; and $\varpi_{j}^{H}\left(t\right)$ denotes the stochastic noise due to modeling residue at time t. The basal level $\delta_{j}^{H}$ denotes the unknown regulations such as methylation. Note that since microRNAs silence gene expression by binding to target mRNAs [39], repression ability $-e_{j\mu }^{H}$ in candidate GRN model should be constrained negative. Likewise, $-\lambda_{j}^{H}$ degradation rate should be restricted non-positive due to the depression regulation of mRNA. Furthermore, though supporting evidence comes from numerous research showing that HBV proteins play a vital role in the regulation of viral and host gene expression, different from other viruses such as HIV, whose viral protein directly binds to nucleic acids [40], HBV proteins are thought to affect host gene expression through transregulation, e.g., transactivation and transrepression [41,42,43,44]. Thus, it seems tenable to exclude the regulations between pathogen proteins and host gene in the Equation (3).

In the same way, for the GRN of pathogen genes in the candidate HPI-GEN, the dynamic regulatory model of the uth pathogen gene can be described as follows:

(4) $$\begin{array}{l} g_{u}^{P}\left(t+1\right)=g_{u}^{P}\left(t\right)+\sum_{\tau =1}^{T_{u}}c_{u\tau }^{P}p_{\tau }^{H}\left(t\right)+\sum_{\ell =1}^{L_{u}}d_{u\ell }^{P}l_{\ell }^{H}\left(t\right)-\sum_{\mu =1}^{M_{u}}e_{u\mu }^{P}g_{u}^{P}\left(t\right)m_{\mu }^{H}\left(t\right)\\ -\lambda_{u}^{P}g_{u}^{P}\left(t\right)+\delta_{u}^{P}+\varpi_{u}^{P}\left(t\right),\;\mathrm{for}\;u=1,2,\ldots ,U,\;-e_{u\mu }^{P}\leq 0\;\mathrm{and}-\lambda_{u}^{P}\leq 0 \end{array}$$

where $g_{u}^{P}\left(t\right),\;p_{\tau }^{H}\left(t\right),\;m_{\mu }^{H}\left(t\right)$ and $l_{\ell }^{H}\left(t\right)$ indicate the expression levels of the uth pathogen gene, the τth host TF, the μth host microRNA, and the ℓth host lncRNA at time t, respectively; $c_{u\tau }^{P},\;-e_{u\mu }^{P}$ and $d_{u\ell }^{P}$ represent the regulation ability of the τth host TF, the μth host microRNA, and the ℓth host lncRNA on the uth pathogen, respectively; $T_{u},\;M_{u}$ and $L_{u}$ denote the number of host TFs, host miRNAs, and host lncRNAs, respectively, which regulate the expression level of the uth pathogen; U indicates the total amount of pathogen genes in GRN; $-\lambda_{u}^{P}$ and $\delta_{u}^{P}$ indicate the degradation rate and the expression basal level of the uth pathogen, respectively; and $\varpi_{u}^{P}\left(t\right)$ denotes the stochastic noise due to modeling residue at time *t*. It is worth noting that there is a consensus on the opinion about the indispensability of host protein synthesis machinery for pathogen protein production and gene transcription [45,46]. Walsh et al. [47] even suggested that viruses are fully reliant on the translation machinery of their host cells to produce the polypeptides that are essential for viral replication. As a result, only gene regulation conducted by host transcription factors $p_{\tau }^{H}\left(t\right)$ instead of pathogen proteins $p_{}^{P}\left(t\right)$ is included in Equation (4).

Since the expression of the μth host miRNA $m_{\mu }^{H}\left(t\right)$ and the ℓ th host $l_{\ell }^{H}\left(t\right)$ in Equation (3) at time *t* might also be regulated by other regulators, the μth miRNA can be described by the following stochastic dynamic regulatory equation:

(5) $$\begin{array}{l} m_{\mu }^{H}\left(t+1\right)=m_{\mu }^{H}\left(t\right)+\sum_{\tau =1}^{T_{\mu }}f_{\mu \tau }^{H}p_{\tau }^{H}\left(t\right)+\sum_{\ell =1}^{L_{\mu }}h_{\mu \ell }^{H}l_{\ell }^{H}\left(t\right)-\sum_{r=1}^{M_{\mu }}k_{\mu r}^{H}m_{\mu }^{H}\left(t\right)m_{r}^{H}\left(t\right)\\ -\varphi_{\mu }^{H}m_{\mu }^{H}\left(t\right)+\eta_{\mu }^{H}+\varepsilon_{\mu }^{H}\left(t\right),\;\mathrm{for}\;\mu =1,2,\ldots ,M,\;-k_{\mu r}^{H}\leq 0\;\mathrm{and}-\varphi_{\mu }^{H}\leq 0 \end{array}$$

where $m_{\mu }^{H}\left(t\right),\;p_{\tau }^{H}\left(t\right),\;m_{r}^{H}\left(t\right)$ and $l_{\ell }^{H}\left(t\right)$ indicate the expression levels of the μth host gene, the τth host TF, the rth host miRNA and the ℓth host lncRNA at time *t*, respectively; $f_{\mu \tau }^{H},\;-k_{\mu r}^{H}$ and $h_{\mu \ell }^{H}$ represent the regulation ability of the τth host TF, the rth host microRNA and the ℓth host lncRNA on the μth host miRNA, respectively; $T_{\mu },\;M_{\mu }$ and $L_{\mu }$ denote the number of host TFs, host miRNAs and host lncRNAs, respectively, which regulate the expression level of the μth host miRNA; M indicates the total number of miRNAs in GRN; $-\varphi_{\mu }^{H}$ and $\eta_{\mu }^{H}$ indicate the degradation rate and the expression basal level of the μth host miRNA, respectively; and $\varepsilon_{\mu }^{H}\left(t\right)$ denotes the stochastic noise due to modeling residue at time *t*.

Along the same line, the dynamic regulatory model of the host lncRNA in candidate GEIN can be described by the following dynamic equation:

(6) $$\begin{array}{l} l_{\ell }^{H}\left(t+1\right)=l_{\ell }^{H}\left(t\right)+\sum_{\tau =1}^{T_{\ell }}x_{\ell \tau }^{H}p_{\tau }^{H}\left(t\right)+\sum_{\ell =1}^{O_{\ell }}y_{\ell o}^{H}l_{o}^{H}\left(t\right)-\sum_{r=1}^{M_{\ell }}z_{\ell \mu }^{H}l_{\ell }^{H}\left(t\right)m_{\mu }^{H}\left(t\right)\\ -\sigma_{\ell }^{H}l_{\ell }^{H}\left(t\right)+\phi_{\ell }^{H}+\psi_{\ell }^{H}\left(t\right),\;\mathrm{for}\;\ell =1,2,\ldots ,L,\;-z_{\ell \mu }^{H}\leq 0\;\mathrm{and}-\sigma_{\ell }^{H}\leq 0 \end{array}$$

where $l_{\ell }^{H}\left(t\right),\;p_{\tau }^{H}\left(t\right),\;m_{\mu }^{H}\left(t\right)$ and $l_{o}^{H}\left(t\right)$ indicate the expression levels of the ℓth host lncRNA, the τth host TF, the μth host microRNA and the ℓth host lncRNA at time t, respectively; $x_{\ell \tau }^{H},\;-z_{\ell \mu }^{H}$ and $y_{\ell o}^{H}$ represent the regulation ability of the τth host TF, the μth host microRNA and the oth host lncRNA on the ℓth host lncRNA, respectively; $T_{\ell },\;M_{\ell }$ and $L_{\ell }$ denote the number of host TFs, host miRNAs and host lncRNAs, respectively, which regulate the expression level of the ℓth host lncRNA; L indicates the total number of lncRNA in GRN; $-\sigma_{\ell }^{H}$ and $\phi_{\ell }^{H}$ indicate the degradation rate and the expression basal level of the ℓth host lncRNA, respectively; and $\psi_{\ell }^{H}\left(t\right)$ denotes the stochastic noise due to modeling residue at time *t*.

### 2.5. Parameter Estimation of the Dynamic Models of Candidate HPI-GEN by System Identification Approach

In order to identify real HPI-GEN, the accurate model parameters training process by executing a system identification approach to the candidate HPI-GEN after the construction of dynamic model Equations (1)–(6) is requisite. Consequently, the stochastic Equations (1) and (2), which depict the relation of protein interactions of the *i*th host and *q*th pathogen protein, can be converted into the linear regression forms as below, respectively:

The *i*th host protein:

(7) $$\begin{array}{l} p_{i}^{H}(t+1)=[p_{i}^{H}p_{1}^{H}(t)\cdots p_{i}^{H}p_{N_{i}}^{H}(t)p_{i}^{H}p_{1}^{P}(t)\;\cdots p_{i}^{H}p_{V_{i}}^{P}(t)g_{i}^{H}(t)p_{i}^{H}(t)1]\left[\begin{array}{c} a_{i1}^{H}\\ \vdots \\ a_{iN_{i}}^{H}\\ b_{i1}^{H}\\ \vdots \\ b_{iV_{i}}^{H}\\ \alpha_{i}^{H}\\ 1-\gamma_{i}^{H}\\ \beta_{i}^{H} \end{array}\right]+v_{i}^{H}(t)\\ \;\;\;\;\;\;≜\phi_{i}^{HP}\left(t\right)\theta_{i}^{HP}+v_{i}^{H}\left(t\right),\;\mathrm{for}\;i=1,2,\ldots ,I \end{array}$$

The *q*th host protein:

(8) $$\begin{array}{l} p_{q}^{P}\left(t+1\right)=[p_{q}^{P}p_{1}^{H}(t)\cdots p_{q}^{P}p_{N_{q}}^{H}(t)p_{q}^{P}p_{1}^{P}(t)\;\cdots p_{q}^{P}p_{V_{q}}^{P}(t)g_{q}^{P}(t)p_{q}^{P}(t)1]\left[\begin{array}{c} a_{q1}^{P}\\ \vdots \\ a_{qN_{q}}^{P}\\ b_{q1}^{H}\\ \vdots \\ b_{qV_{q}}^{P}\\ \alpha_{q}^{P}\\ 1-\gamma_{q}^{P}\\ \beta_{q}^{P} \end{array}\right]+v_{q}^{P}(t)\\ \;\;\;\;\;≜\phi_{q}^{PP}\left(t\right)\theta_{q}^{PP}+v_{q}^{P}\left(t\right),\;\mathrm{for}\;q=1,2,\ldots ,Q \end{array}$$

where $\phi_{i}^{HP}\left(t\right)$ and $\phi_{q}^{PP}\left(t\right)$ represent the regression vectors that can be obtained from the expression data and $\theta_{i}^{HP}$ and $\theta_{q}^{PP}$ are the unknown parameter vectors to be estimated for the ith host protein, qth pathogen protein in host PPIN, respectively.

Thence, the Equations (7) and (8) of the *i*th host and *q*th pathogen protein can further be augmented for $Y_{i\;}$ and $Y_{q\;}$ time points as the following forms, respectively:

The *i*th host protein:

(9) $$\left[\begin{array}{c} p_{i}^{H}\left(t_{2}\right)\\ p_{i}^{H}\left(t_{3}\right)\\ \vdots \\ p_{i}^{H}\left(t_{Y_{i}}+1\right) \end{array}\right]=\left[\begin{array}{c} \phi_{i}^{HP}\left(t_{1}\right)\\ \phi_{i}^{HP}\left(t_{2}\right)\\ \vdots \\ \phi_{i}^{HP}\left(t_{Y_{i}}\right) \end{array}\right]\theta_{i}^{HP}+\left[\begin{array}{c} v_{i}^{H}\left(t_{1}\right)\\ v_{i}^{H}\left(t_{2}\right)\\ \vdots \\ v_{i}^{H}\left(t_{Y_{i}}\right) \end{array}\right],\;\mathrm{for}\;i=1,2,\ldots ,I$$

the Equation (9) can also be simply represented as

(10) $$P_{i}^{H}=\Phi_{i}^{HP}\theta_{i}^{HP}+\Omega_{i}^{HP},\mathrm{for}\;i=1,2,\ldots ,I$$

The *q*th host protein:

(11) $$\left[\begin{array}{c} p_{q}^{P}\left(t_{2}\right)\\ p_{q}^{P}\left(t_{3}\right)\\ \vdots \\ p_{q}^{P}\left(t_{Y_{q}}+1\right) \end{array}\right]=\left[\begin{array}{c} \phi_{q}^{PP}\left(t_{1}\right)\\ \phi_{q}^{PP}\left(t_{2}\right)\\ \vdots \\ \phi_{q}^{PP}\left(t_{Y_{q}}\right) \end{array}\right]\theta_{q}^{PP}+\left[\begin{array}{c} v_{q}^{P}\left(t_{1}\right)\\ v_{q}^{P}\left(t_{2}\right)\\ \vdots \\ v_{q}^{P}\left(t_{Y_{q}}\right) \end{array}\right],\;\mathrm{for}\;q=1,2,\ldots ,Q$$

the Equation (11) can also be simply represented as

(12) $$P_{q}^{P}=\Phi_{q}^{PP}\theta_{q}^{PP}+\Omega_{q}^{PP},\mathrm{for}\;q=1,2,\ldots ,Q$$

After a succession of equation transformation procedure in advance, the parameters in vectors $\theta_{i}^{HP}$ and $\theta_{q}^{PP}$ of Equations (10) and (12) can be estimated by individually employing the following constrained least-squares estimation problems, $\theta_{i}^{HP}$ parameters estimation:

(13) $$\begin{array}{l} \underset{\theta_{i}^{HP}}{{\hat{\theta }}_{i}^{HP}=\min}{\left\|\Phi_{i}^{HP}\theta_{i}^{HP}-P_{i}^{H}\right\|}_{2}^{2},\;\mathrm{subject}\;\mathrm{to}\;A_{i}^{HP}{\hat{\theta }}_{i}^{HP}\leq b_{i}^{HP}\\ \mathrm{where}\;A_{i}^{HP}=\left[\begin{array}{ccccccccc} 0 & \cdots & 0 & 0 & \cdots & 0 & -1 & 0 & 0\\ 0 & \cdots & 0 & 0 & \cdots & 0 & 0 & 1 & 0 \end{array}\right]\in {\mathbb{R}}^{2\times (I+Q+3)}\;\mathrm{and}\;b_{i}^{HP}=\left[\begin{array}{c} 0\\ 1 \end{array}\right] \end{array}$$

$\theta_{q}^{PP}$ parameters estimation:

(14) $$\begin{array}{l} {\hat{\theta }}_{q}^{PP}=\underset{\theta_{q}^{PP}}{\min}{\left\|\Phi_{q}^{PP}\theta_{q}^{PP}-P_{q}^{P}\right\|}_{2}^{2},\;\mathrm{subject}\;\mathrm{to}\;A_{q}^{PP}{\hat{\theta }}_{q}^{PP}\leq b_{q}^{PP}\\ \mathrm{where}\;A_{q}^{PP}=\left[\begin{array}{ccccccccc} 0 & \cdots & 0 & 0 & \cdots & 0 & -1 & 0 & 0\\ 0 & \cdots & 0 & 0 & \cdots & 0 & 0 & 1 & 0 \end{array}\right]\in {\mathbb{R}}^{2\times (I+Q+3)}\;\mathrm{and}\;b_{q}^{PP}=\left[\begin{array}{c} 0\\ 1 \end{array}\right] \end{array}$$

Subsequently, we can acquire the interaction parameters in PPIN Equations (1) and (2) individually by resolving the least-squares problems in (13) and (14) with the help of the function *lsqlin* in MATLAB optimization toolbox and simultaneously ensure the protein translation rate $\alpha_{i}^{H}$ and $\alpha_{q}^{P}$ to be a non-negative value and the protein degradation rate $-\gamma_{i}^{H}$ and $-\gamma_{q}^{P}$ to be a non-positive value; that is to say $\alpha_{i}^{H},\alpha_{q}^{P}\geq 0$ and $-\gamma_{i}^{H},-\gamma_{q}^{P}\leq 0$.

In the same manner, the dynamic Equations (3)–(6) which depict the relationship of gene regulations for the *j*th host gene, *u*th pathogen gene, μth host miRNA, and ℓth host lncRNA can be rewritten into the linear regression forms below, respectively:

The *j*th host gene:

(15) $$\begin{array}{l} g_{j}^{H}\left(t+1\right)=[p_{1}^{H}(t)\cdots p_{T_{j}}^{H}(t)g_{j}^{H}(t)m_{1}^{H}(t)\cdots g_{j}^{H}(t)m_{M_{j}}^{H}(t)\;l_{1}^{H}(t)\cdots l_{L_{j}}^{H}(t)\;g_{j}^{H}(t)\;1]\left[\begin{array}{c} c_{j1}^{H}\\ \vdots \\ c_{jT_{j}}^{H}\\ -e_{j1}^{H}\\ \vdots \\ -e_{jM_{j}}^{H}\\ d_{j1}^{H}\\ \vdots \\ d_{jL_{j}}^{H}\\ 1-\lambda_{j}^{H}\\ \delta_{j}^{H} \end{array}\right]+\varpi_{j}^{H}(t)\\ \;\;\;\;\;≜\phi_{j}^{HG}\left(t\right)\theta_{j}^{HG}+\varpi_{j}^{H}\left(t\right),\;\mathrm{for}\;j=1,2,\ldots ,J \end{array}$$

The *u*th pathogen gene:

(16) $$\begin{array}{l} g_{u}^{P}\left(t+1\right)=[p_{1}^{H}(t)\cdots p_{T_{u}}^{H}(t)g_{u}^{P}(t)m_{1}^{H}(t)\cdots g_{u}^{P}(t)m_{M_{u}}^{H}(t)\;l_{1}^{H}(t)\cdots l_{L_{u}}^{H}(t)\;g_{u}^{P}(t)\;1]\left[\begin{array}{c} c_{u1}^{P}\\ \vdots \\ c_{uT_{u}}^{P}\\ -e_{u1}^{P}\\ \vdots \\ -e_{uM_{u}}^{P}\\ d_{u1}^{P}\\ \vdots \\ d_{uL_{u}}^{P}\\ 1-\lambda_{u}^{P}\\ \delta_{u}^{P} \end{array}\right]+\varpi_{u}^{P}(t)\\ \;\;\;\;\;≜\phi_{u}^{PG}\left(t\right)\theta_{u}^{PG}+\varpi_{u}^{P}\left(t\right),\;\mathrm{for}\;u=1,2,\ldots ,U \end{array}$$

The μth host miRNA:

(17) $$\begin{array}{l} m_{\mu }^{H}\left(t+1\right)=[p_{1}^{H}(t)\cdots p_{T_{\mu }}^{H}m_{\mu }^{H}(t)m_{1}^{H}(t)\cdots m_{\mu }^{H}(t)m_{R_{\mu }}^{H}(t)\;l_{1}^{H}(t)\cdots l_{L_{\mu }}^{H}(t)\;m_{\mu }^{H}(t)\;1]\left[\begin{array}{c} f_{\mu 1}^{H}\\ \vdots \\ f_{\mu T_{\mu }}^{H}\\ -k_{\mu 1}^{H}\\ \vdots \\ -k_{\mu R_{\mu }}^{H}\\ h_{\mu 1}^{H}\\ \vdots \\ h_{\mu L_{\mu }}^{H}\\ 1-\varphi_{\mu }^{H}\\ \eta_{\mu }^{H} \end{array}\right]+\varepsilon_{\mu }^{H}(t)\\ \;\;\;\;\;≜\phi_{\mu }^{HM}\left(t\right)\theta_{\mu }^{HM}+\varepsilon_{\mu }^{H}\left(t\right),\;\mathrm{for}\;\mu =1,2,\ldots ,M \end{array}$$

The ℓth host lncRNA:

(18) $$\begin{array}{l} l_{\ell }^{H}\left(t+1\right)=[p_{1}^{H}(t)\cdots p_{T_{\ell }}^{H}l_{\ell }^{H}(t)m_{1}^{H}(t)\cdots l_{\ell }^{H}(t)m_{M_{\ell }}^{H}(t)\;l_{1}^{H}(t)\cdots l_{L_{\ell }}^{H}(t)\;l_{\ell }^{H}(t)\;1]\left[\begin{array}{c} x_{\ell 1}^{H}\\ \vdots \\ x_{\ell T_{\ell }}^{H}\\ -z_{\ell 1}^{H}\\ \vdots \\ -z_{\ell M_{\ell }}^{H}\\ y_{\ell 1}^{H}\\ \vdots \\ y_{\ell L_{\ell }}^{H}\\ 1-\sigma_{\ell }^{H}\\ \phi_{\ell }^{H} \end{array}\right]+\psi_{\ell }^{H}(t)\\ \;\;\;\;\;≜\phi_{\ell }^{HL}\left(t\right)\theta_{\ell }^{HL}+\psi_{\ell }^{H}\left(t\right),\;\mathrm{for}\;\ell =1,2,\ldots L \end{array}$$

where $\phi_{j}^{HG}\left(t\right),\;\phi_{u}^{PG}\left(t\right),\;\phi_{\mu }^{HM}\left(t\right)$ and $\phi_{\ell }^{HL}\left(t\right)$ represent the regression vectors that can be obtained from the expression data and $\theta_{j}^{HG},\;\theta_{u}^{PG},\;\theta_{\mu }^{HM}$ and $\theta_{\ell }^{HL}$ are the unknown parameter vectors to be estimated for the jth host gene, uth pathogen gene, μth host miRNA, and ℓth host lncRNA in host GRN, respectively.

Then, Equations (15)–(18) of the *j*th host gene, *u*th pathogen gene, μth host miRNA, and ℓth host lncRNA can be further augmented for $Y_{j},\;Y_{u},\;Y_{\mu }$ and $Y_{\ell }$ time points as the following forms, respectively:

The *j*th host gene:

(19) $$\left[\begin{array}{c} g_{j}^{H}(t_{2})\\ g_{j}^{H}(t_{3})\\ \vdots \\ g_{j}^{H}(t_{Y_{j}}+1) \end{array}\right]=\left[\begin{array}{c} \phi_{j}^{HG}(t_{1})\\ \phi_{j}^{HG}(t_{2})\\ \vdots \\ \phi_{j}^{HG}(t_{Y_{j}}) \end{array}\right]\theta_{j}^{HG}+\left[\begin{array}{c} \varpi_{j}^{H}(t_{1})\\ \varpi_{j}^{H}(t_{2})\\ \vdots \\ \varpi_{j}^{H}(t_{Y_{j}}) \end{array}\right],\;\mathrm{for}\;j=1,2,\ldots ,J$$

the Equation (19) can also be simply represented as:

(20) $$G_{j}^{H}=\Phi_{j}^{HG}\theta_{j}^{HG}+\Omega_{j}^{HG},\;\mathrm{for}\;j=1,2,\ldots ,J$$

The *u*th pathogen gene:

(21) $$\left[\begin{array}{c} g_{u}^{P}(t_{2})\\ g_{u}^{P}(t_{3})\\ \vdots \\ g_{u}^{P}(t_{Y_{u}}+1) \end{array}\right]=\left[\begin{array}{c} \phi_{u}^{PG}(t_{1})\\ \phi_{u}^{PG}(t_{2})\\ \vdots \\ \phi_{u}^{PG}(t_{Y_{u}}) \end{array}\right]\theta_{u}^{HG}+\left[\begin{array}{c} \varpi_{u}^{P}(t_{1})\\ \varpi_{u}^{P}(t_{2})\\ \vdots \\ \varpi_{u}^{P}(t_{Y_{u}}) \end{array}\right],\;\mathrm{for}\;u=1,2,\ldots ,U$$

the Equation (21) can also be simply represented as:

(22) $$G_{u}^{P}=\Phi_{u}^{PG}\theta_{u}^{PG}+\Omega_{u}^{PG},\;\mathrm{for}\;u=1,2,\ldots ,U$$

The μth host miRNA:

(23) $$\left[\begin{array}{c} m_{\mu }^{H}(t_{2})\\ m_{\mu }^{H}(t_{3})\\ \vdots \\ m_{\mu }^{H}(t_{Y_{\mu }}+1) \end{array}\right]=\left[\begin{array}{c} \phi_{\mu }^{HM}(t_{1})\\ \phi_{\mu }^{HM}(t_{2})\\ \vdots \\ \phi_{\mu }^{HM}(t_{Y_{\mu }}) \end{array}\right]\theta_{\mu }^{HM}+\left[\begin{array}{c} \varepsilon_{j}^{H}(t_{1})\\ \varepsilon_{j}^{H}(t_{2})\\ \vdots \\ \varepsilon_{j}^{H}(t_{Y_{\mu }}) \end{array}\right],\;\mathrm{for}\;\mu =1,2,\ldots ,M$$

the Equation (23) can also be simply represented as:

(24) $$M_{\mu }^{H}=\Phi_{\mu }^{HM}\theta_{\mu }^{HM}+\Omega_{\mu }^{HM},\;\mathrm{for}\;\mu =1,2,\ldots ,M$$

The ℓth host lncRNA:

(25) $$\left[\begin{array}{c} l_{\ell }^{H}(t_{2})\\ l_{\ell }^{H}(t_{3})\\ \vdots \\ l_{\ell }^{H}(t_{Y_{\ell }}+1) \end{array}\right]=\left[\begin{array}{c} \phi_{\ell }^{HL}(t_{1})\\ \phi_{\ell }^{HL}(t_{2})\\ \vdots \\ \phi_{\ell }^{HL}(t_{Y_{\ell }}) \end{array}\right]\theta_{j}^{HG}+\left[\begin{array}{c} \psi_{\ell }^{H}(t_{1})\\ \psi_{\ell }^{H}(t_{2})\\ \vdots \\ \psi_{\ell }^{H}(t_{Y_{\ell }}) \end{array}\right],\;\mathrm{for}\;\ell =1,2,\ldots ,L$$

the Equation (25) can also be simply represented as:

(26) $$L_{\ell }^{H}=\Phi_{\ell }^{HG}\theta_{\ell }^{HG}+\Omega_{\ell }^{HG},\;\mathrm{for}\;\ell =1,2,\ldots ,L$$

After a succession of equation transformation procedure in advance, the parameters in vectors $\theta_{j}^{HG},\;\theta_{u}^{PG},\;\theta_{\mu }^{HM}$ and $\theta_{\ell }^{HL}$ of Equations (20), (22), (24) and (26) can be estimated by individually employing the following constrained least-square estimation problem,

(27) $$\begin{array}{l} {\hat{\theta }}_{j}^{HG}=\underset{\theta_{j}^{HG}}{\min}\frac{1}{2}{\left\|\;\Phi_{j}^{HG}\theta_{j}^{HG}-G_{j}^{H}\right\|}_{2}^{2},\;subject\;to\;A_{j}^{HG}{\hat{\theta }}_{j}^{HG}\leq b_{j}^{HG}\;where\;b_{j}^{HG}=\left[\begin{array}{c} 0\\ 1 \end{array}\right]\;\\ {\mathrm{and}\;A}_{j}^{HG}=\left[\begin{array}{cccccccccccccc} 0 & 0 & \cdots & 0 & 1 & 0 & \cdots & 0 & 0 & 0 & \cdots & 0 & 0 & 0\\ 0 & 0 & \cdots & 0 & 0 & 1 & \cdots & 0 & 0 & 0 & \cdots & 0 & 0 & 0\\ \vdots & \vdots & \ddots & \vdots & \vdots & \vdots & \ddots & \vdots & \vdots & \vdots & \ddots & \vdots & \vdots & \vdots \\ 0 & 0 & \cdots & 0 & 0 & 0 & \cdots & 1 & 0 & 0 & \cdots & 0 & 0 & 0\\ 0 & 0 & \cdots & 0 & 0 & 0 & \cdots & 0 & 0 & 0 & \cdots & 0 & 1 & 0 \end{array}\right]\in {\mathbb{R}}^{\left(M_{j}+1)\times (T_{j}+M_{j}+L_{j}+2\right)}\; \end{array}$$

(28) $$\begin{array}{l} {\hat{\theta }}_{u}^{PG}=\underset{\theta_{u}^{PG}}{\min}\frac{1}{2}{\left\|\;\Phi_{u}^{PG}\theta_{u}^{PG}-G_{u}^{P}\right\|}_{2}^{2},\;subject\;to\;A_{u}^{PG}{\hat{\theta }}_{u}^{PG}\leq b_{u}^{PG}\;where\;b_{u}^{PG}=\left[\begin{array}{c} 0\\ 1 \end{array}\right]\;\\ {\mathrm{and}\;A}_{u}^{PG}=\left[\begin{array}{cccccccccccccc} 0 & 0 & \cdots & 0 & 1 & 0 & \cdots & 0 & 0 & 0 & \cdots & 0 & 0 & 0\\ 0 & 0 & \cdots & 0 & 0 & 1 & \cdots & 0 & 0 & 0 & \cdots & 0 & 0 & 0\\ \vdots & \vdots & \ddots & \vdots & \vdots & \vdots & \ddots & \vdots & \vdots & \vdots & \ddots & \vdots & \vdots & \vdots \\ 0 & 0 & \cdots & 0 & 0 & 0 & \cdots & 1 & 0 & 0 & \cdots & 0 & 0 & 0\\ 0 & 0 & \cdots & 0 & 0 & 0 & \cdots & 0 & 0 & 0 & \cdots & 0 & 1 & 0 \end{array}\right]\in {\mathbb{R}}^{\left(M_{u}+1)\times (T_{u}+M_{u}+L_{u}+2\right)}\; \end{array}$$

(29) $$\begin{array}{l} {\hat{\theta }}_{\mu }^{HM}=\underset{\theta_{\mu }^{HM}}{\min}\frac{1}{2}{\left\|\;\Phi_{\mu }^{HM}\theta_{\mu }^{HM}-M_{\mu }^{H}\right\|}_{2}^{2},\;subject\;to\;A_{\mu }^{HM}{\hat{\theta }}_{\mu }^{HM}\leq b_{\mu }^{HM}\;where\;b_{\mu }^{HM}=\left[\begin{array}{c} 0\\ 1 \end{array}\right]\;\\ {\mathrm{and}\;A}_{\mu }^{HM}=\left[\begin{array}{cccccccccccccc} 0 & 0 & \cdots & 0 & 1 & 0 & \cdots & 0 & 0 & 0 & \cdots & 0 & 0 & 0\\ 0 & 0 & \cdots & 0 & 0 & 1 & \cdots & 0 & 0 & 0 & \cdots & 0 & 0 & 0\\ \vdots & \vdots & \ddots & \vdots & \vdots & \vdots & \ddots & \vdots & \vdots & \vdots & \ddots & \vdots & \vdots & \vdots \\ 0 & 0 & \cdots & 0 & 0 & 0 & \cdots & 1 & 0 & 0 & \cdots & 0 & 0 & 0\\ 0 & 0 & \cdots & 0 & 0 & 0 & \cdots & 0 & 0 & 0 & \cdots & 0 & 1 & 0 \end{array}\right]\in {\mathbb{R}}^{\left(M_{\mu }+1)\times (T_{\mu }+M_{\mu }+L_{\mu }+2\right)}\; \end{array}$$

(30) $$\begin{array}{l} {\hat{\theta }}_{\ell }^{HL}=\underset{\theta_{\ell }^{HL}}{\min}\frac{1}{2}{\left\|\;\Phi_{\ell }^{HL}\theta_{\ell }^{HL}-L_{\ell }^{H}\right\|}_{2}^{2},\;subject\;to\;A_{\ell }^{HL}{\hat{\theta }}_{\ell }^{HL}\leq b_{\ell }^{HL}\;whereb_{\ell }^{HL}=\left[\begin{array}{c} 0\\ 1 \end{array}\right]\;\\ {\mathrm{and}\;A}_{\ell }^{HL}=\left[\begin{array}{cccccccccccccc} 0 & 0 & \cdots & 0 & 1 & 0 & \cdots & 0 & 0 & 0 & \cdots & 0 & 0 & 0\\ 0 & 0 & \cdots & 0 & 0 & 1 & \cdots & 0 & 0 & 0 & \cdots & 0 & 0 & 0\\ \vdots & \vdots & \ddots & \vdots & \vdots & \vdots & \ddots & \vdots & \vdots & \vdots & \ddots & \vdots & \vdots & \vdots \\ 0 & 0 & \cdots & 0 & 0 & 0 & \cdots & 1 & 0 & 0 & \cdots & 0 & 0 & 0\\ 0 & 0 & \cdots & 0 & 0 & 0 & \cdots & 0 & 0 & 0 & \cdots & 0 & 1 & 0 \end{array}\right]\in {\mathbb{R}}^{\left(M_{\ell }+1)\times (T_{\ell }+M_{\ell }+L_{\ell }+2\right)}\; \end{array}$$

Through applying the function lsqlin in MATLAB optimization toolbox to solve the constrained least-squares estimation problem in (27)–(30), we could obtain optimal regulatory parameters for GRN Equations in (3)–(6) and concurrently guarantee that the miRNA repression ability $-e_{j\mu }^{H},\;-e_{u\mu }^{P},\;-k_{\mu r}^{H}$ and $-z_{\ell \mu }^{H}$ as well as the degradation rate $-\lambda_{j}^{H},\;-\lambda_{u}^{P},\;-\varphi_{\mu }^{H}$ and $-\sigma_{\ell }^{H}$ values corresponding to the *j*th host gene, the *u*th pathogen gene, the μth host miRNA, and the ℓth host lncRNA to be non-positive, respectively. 

So far, the structure of dynamic regression model construction is complete, yet if we substitute the expression data into the linear model right away, infinite solutions problems will emerge due to the lack of enough data points, in other words, information deficiency in comparison with parameters for evaluation, that is, overfitting. Thus, the cubic spline for extra numbers of time-profile data points interpolation is applied to prevent this trouble (For more details, readers can refer to Appendix A). Then, with the processed RNA-seq data, the accurate solutions of the constrained least-squares estimation problems in (13)–(14) and (27)–(30) can be attained.

Moreover, since the measurement technology of genome-wide protein expression in HepaRG cells and Hepatitis B Virus during infection hasn’t been realized yet, and supporting evidence comes from research showing that the cellular concentrations of proteins correlate with the abundances of their corresponding mRNAs, which implied that the variance in protein abundance can be explained by that of mRNA [48,49,50,51] and a correlation coefficient of 48% was also obtained between the mRNA and protein abundances in human liver [49], i.e., the RNA-seq data of gene expressions can substitute protein expressions and contribute to sufficient information for resolving above constrained least-squares parameter estimation problems in (13) and (14) and (27)–(30). One could retain more information in our previous research [52].

### 2.6. Determination of Significant Interaction Pairs

On account of approaching the best combination to constitute the robust HPI-GEN in the parameter identification procedures, we exploited the cost function to obtain the optimal fit for the host/pathogen RNA-seq data, to be more precisely, estimation of the expected relative distance between the fitted model and the unrecognized authentic molecular pathogenic mechanism that actually generated the observed data. On the whole, as the cost function of the parameter estimation for linear regression model, mean squared error (MSE) is enough to calculate the residual variance. However, the cost from the model complexity that might also influences the performance should be taken into consideration as well. Akaike Information Criterion (AIC), taking the place of MSE, was thereby selected to assess the residual variance and model complexity at once. As the expected residual variance declines with rising parameter numbers for inadequate model complexities, there should be a minimum around the correct parameter number after repeated coordination [52]. Meanwhile, owing to computational efficiency, computing the AIC statistics for all possible combinations is impracticable. Stepwise methods are developed to decreasing the complexity of exhaustive searching [52]. Finally, while reaching the minimum of AIC value, we could acquire the real number of interactions or regulations for each protein (gene) one by one in the candidate HPI-GEN. Those insignificant regulations or interactions out of the real number determined by AIC should be pruned to obtain the real HPI-GEN.

For each model composing PPIN, the AIC values of the ith host and the qth pathogen protein can be defined individually as the following equations:

The ith host protein:

(31) $$\begin{array}{ll} AIC_{i}^{HP}\left(N_{i},V_{i}\right) & =\log\left(\frac{1}{Y_{i}}{\left(P_{i}^{H}-\Phi_{i}^{HP}{\hat{\theta }}_{i}^{HP}\right)}^{T}\left(P_{i}^{H}-\Phi_{i}^{HP}{\hat{\theta }}_{i}^{HP}\right)\right)+\frac{2\left(N_{i}+V_{i}\right)}{Y_{i}}\\ & =\log\left[{\left(\sigma_{i}^{HP}\right)}^{2}\;\right]+\frac{2\left(N_{i}+V_{i}\right)}{Y_{i}} \end{array}$$

The qth pathogen protein:

(32) $$\begin{array}{ll} AIC_{q}^{PP}\left(N_{q},V_{q}\right) & =\log\left(\frac{1}{Y_{q}}{\left(P_{q}^{P}-\Phi_{q}^{PP}{\hat{\theta }}_{q}^{PP}\right)}^{T}\left(P_{q}^{P}-\Phi_{q}^{PP}{\hat{\theta }}_{q}^{PP}\right)\right)+\frac{2\left(N_{q}+V_{q}\right)}{Y_{q}}\\ & =\log\left[{\left(\sigma_{q}^{PP}\right)}^{2}\;\right]+\frac{2\left(N_{q}+V_{q}\right)}{Y_{q}} \end{array}$$

for i=1,…,I and q=1,…,Q, where $AIC_{i}^{HP}\left(N_{i},V_{i}\right)$ with the model complexity $N_{i}+V_{i}$ and $AIC_{q}^{PP}\left(N_{q},V_{q}\right)$ with the model complexity $N_{q}+V_{q}$ denote the ith host and the qth pathogen protein in PPI model, respectively; ${\left(\sigma_{i}^{HP}\right)}^{2}$ and ${\left(\sigma_{q}^{PP}\right)}^{2}$ signify the covariance of estimated residual error between $P_{i}^{H}$ and $\Phi_{i}^{HP}{\hat{\theta }}_{i}^{HP}$ and between $P_{q}^{P}$ and $\Phi_{q}^{PP}{\hat{\theta }}_{q}^{PP}$, respectively; and ${\hat{\theta }}_{i}^{HP}$ and ${\hat{\theta }}_{q}^{PP}$ represent the estimated parameters acquired from the solutions of the parameter estimation problems in (13) and (14), respectively. Suppose $N_{i}^{*},V_{i}^{*}$ and $N_{q}^{*},V_{q}^{*}$ could minimize $AIC_{i}^{HP}\left(N_{i},V_{i}\right)$ and $AIC_{q}^{PP}\left(N_{q},V_{q}\right)$ in (31) and (32), respectively. Then, $N_{i}^{*},V_{i}^{*}$ and $N_{q}^{*},V_{q}^{*}$ denote the real number of PPIs in the *i*th host protein and the *q*th pathogen protein. The insignificant PPIs out of these numbers should be pruned as false positives from candidate PPIs to obtain real PPIs in HPI-GEN. 

For each model comprising GRN, the AIC values of the *j*th host gene, the *u*th pathogen gene, the μth host miRNA, and the ℓth host lncRNA can be defined individually as following equations:

The *j*th host gene:

(33) $$\begin{array}{ll} AIC_{j}^{HG}\left(T_{j}+M_{j}+L_{j}\right) & =\log\left(\frac{1}{Y_{j}}{\left(G_{j}^{H}-\Phi_{j}^{HG}{\hat{\theta }}_{j}^{HG}\right)}^{T}\left(G_{j}^{H}-\Phi_{j}^{HG}{\hat{\theta }}_{j}^{HG}\right)\right)+\frac{2\left(T_{j}+M_{j}+L_{j}\right)}{Y_{j}}\\ & =\log\left[{\left(\sigma_{j}^{HG}\right)}^{2}\;\right]+\frac{2\left(T_{j}+M_{j}+L_{j}\right)}{Y_{j}} \end{array}$$

The *u*th pathogen gene:

(34) $$\begin{array}{ll} AIC_{u}^{PG}\left(T_{u}+M_{u}+L_{u}\right) & =\log\left(\frac{1}{Y_{u}}{\left(G_{u}^{P}-\Phi_{u}^{PG}{\hat{\theta }}_{u}^{PG}\right)}^{T}\left(G_{u}^{P}-\Phi_{u}^{PG}{\hat{\theta }}_{u}^{PG}\right)\right)+\frac{2\left(T_{u}+M_{u}+L_{u}\right)}{Y_{u}}\\ & =\log\left[{\left(\sigma_{u}^{PG}\right)}^{2}\;\right]+\frac{2\left(T_{u}+M_{u}+L_{u}\right)}{Y_{u}} \end{array}$$

The μth host miRNA:

(35) $$\begin{array}{ll} AIC_{\mu }^{HM}\left(T_{\mu }+M_{\mu }+L_{\mu }\right) & =\log\left(\frac{1}{Y_{\mu }}{\left(M_{\mu }^{H}-\Phi_{\mu }^{HM}{\hat{\theta }}_{\mu }^{HM}\right)}^{T}\left(M_{\mu }^{H}-\Phi_{\mu }^{HM}{\hat{\theta }}_{\mu }^{HM}\right)\right)+\frac{2\left(T_{\mu }+M_{\mu }+L_{\mu }\right)}{Y_{\mu }}\\ & =\log\left[{\left(\sigma_{\mu }^{HM}\right)}^{2}\;\right]+\frac{2\left(T_{\mu }+M_{\mu }+L_{\mu }\right)}{Y_{\mu }} \end{array}$$

The ℓth host lncRNA:

(36) $$\begin{array}{ll} AIC_{\ell }^{HL}\left(T_{\ell }+M_{\ell }+L_{\ell }\right) & =\log\left(\frac{1}{Y_{\ell }}{\left(L_{\ell }^{H}-\Phi_{\ell }^{HL}{\hat{\theta }}_{\ell }^{HL}\right)}^{T}\left(L_{\ell }^{H}-\Phi_{\ell }^{HL}{\hat{\theta }}_{\ell }^{HL}\right)\right)+\frac{2\left(T_{\ell }+M_{\ell }+L_{\ell }\right)}{Y_{\ell }}\\ & =\log\left[{\left(\sigma_{\ell }^{HL}\right)}^{2}\;\right]+\frac{2\left(T_{\ell }+M_{\ell }+L_{\ell }\right)}{Y_{\ell }} \end{array}$$

for j=1,…,J, q=1,…,Q, μ=1,…,M, and ℓ=1,…,L where $AIC_{j}^{HG}\left(T_{j}+M_{j}+L_{j}\right),\;AIC_{u}^{PG}\left(T_{u}+M_{u}+L_{u}\right),\;AIC_{\mu }^{HM}\left(T_{\mu }+M_{\mu }+L_{\mu }\right)$ and $AIC_{\ell }^{HL}\left(T_{\ell }+M_{\ell }+L_{\ell }\right)$ with the model complexity $T_{j}+M_{j}+L_{j},\;T_{u}+M_{u}+L_{u},\;T_{\mu }+M_{\mu }+L_{\mu }$ and $T_{\ell }+M_{\ell }+L_{\ell }$ denote the AIC values of the *j*th host gene, the *u*th pathogen gene, the μth host miRNA, and the ℓth host lncRNA in GRN model, respectively; ${\left(\sigma_{j}^{HG}\right)}^{2},\;{\left(\sigma_{u}^{PG}\right)}^{2},\;{\left(\sigma_{\mu }^{HM}\right)}^{2}$ and ${\left(\sigma_{\ell }^{HL}\right)}^{2}$ signify the covariance of estimated residual error between $G_{j}^{H}$ and $\Phi_{j}^{HG}{\hat{\theta }}_{j}^{HG},$ $G_{u}^{P}$ and $\Phi_{u}^{PG}{\hat{\theta }}_{u}^{PG},$ $M_{\mu }^{H}$ and $\Phi_{\mu }^{HM}{\hat{\theta }}_{\mu }^{HM},$ and $L_{\ell }^{H}$ and $\Phi_{\ell }^{HL}{\hat{\theta }}_{\ell }^{HL},$ respectively; and ${\hat{\theta }}_{j}^{HG},\;{\hat{\theta }}_{u}^{PG},\;{\hat{\theta }}_{\mu }^{HM}$ and ${\hat{\theta }}_{\ell }^{HL}$ represent the estimated parameters acquired from the solutions of the parameter estimation problems in (27)–(30), respectively. Similarly, the real numbers of regulation by TF, miRNA, lncRNA are obtained by minimizing the corresponding AICs in (33)–(36). The insignificant regulations out of these real numbers should be pruned as false positives to obtain the real regulations by TF, miRNA, lncRNA in HPI-GEN.

So far, through a sequence of systematic identification processes, the construction for real HPI-GENs is broadly accomplished by pruning those insignificant interactions and regulations out of the corresponding AIC. Unfortunately, due to the excessively enormous network architecture, we could hardly extract the core pathways to investigate the pathogenesis during HBV infection, let alone screening of candidate proteins as new targets for drug development. We thereby extracted the core network from the real HPI-GEN via applying the principal network projection (PNP) method.

### 2.7. Extracting Core Network Structure from the Real HPI-GEN by Using PNP Approach

Prior to core network extraction from the real HPI-GEN through applying PNP method, it is essential to integrate the network estimated parameters from PPIN and GRN into a system matrix A as follows,

$$\begin{array}{ll} A & =\;\left[\begin{array}{llll} A_{HP,HP} & A_{HP,PP} & \;0 & 0\\ A_{PP,HP} & A_{PP,PP} & 0 & 0\\ A_{HG,HP} & \;0 & A_{HG,HM} & A_{HG,HL}\\ A_{HM,HP} & \;0 & A_{HM,HM} & A_{HM,HL}\\ A_{HL,HP} & \;0 & A_{HL,HM} & A_{HL,HL}\\ A_{PG,HP} & \;0 & A_{PG,HM} & A_{PG,HL} \end{array}\right]\\ & \;=\left[\begin{array}{cccc} \;\begin{array}{ccc} {\hat{a}}_{11}^{H} & \cdots & {\hat{a}}_{1I}^{H}\\ \vdots & {\hat{a}}_{in}^{H} & \vdots \\ {\hat{a}}_{I1}^{H} & \cdots & {\hat{a}}_{II}^{H} \end{array} & \begin{array}{ccc} {\hat{b}}_{11}^{H} & \cdots & {\hat{b}}_{1Q}^{H}\\ \vdots & {\hat{b}}_{iv}^{H} & \vdots \\ {\hat{b}}_{I1}^{H} & \cdots & {\hat{b}}_{IQ}^{H} \end{array} & \begin{array}{ccc} 0 & \cdots & 0\\ \vdots & 0 & \vdots \\ 0 & \cdots & 0 \end{array} & \begin{array}{ccc} 0 & \cdots & 0\\ \vdots & 0 & \vdots \\ 0 & \cdots & 0 \end{array}\\ \;\begin{array}{ccc} {\hat{a}}_{11}^{P} & \cdots & {\hat{a}}_{1I}^{P}\\ \vdots & {\hat{a}}_{qn}^{P} & \vdots \\ {\hat{a}}_{Q1}^{P} & \cdots & {\hat{a}}_{QI}^{P} \end{array} & \begin{array}{ccc} {\hat{b}}_{11}^{P} & \cdots & {\hat{b}}_{1Q}^{P}\\ \vdots & {\hat{b}}_{qv}^{P} & \vdots \\ {\hat{b}}_{Q1}^{P} & \cdots & {\hat{b}}_{QQ}^{P} \end{array} & \begin{array}{ccc} 0 & \cdots & 0\\ \vdots & 0 & \vdots \\ 0 & \cdots & 0 \end{array} & \;\begin{array}{ccc} 0 & \cdots & 0\\ \vdots & 0 & \vdots \\ 0 & \cdots & 0 \end{array}\\ \;\begin{array}{ccc} {\hat{c}}_{11}^{H} & \cdots & {\hat{c}}_{1I}^{H}\\ \vdots & {\hat{c}}_{j\tau }^{H} & \vdots \\ {\hat{c}}_{I1}^{H} & \cdots & {\hat{c}}_{II}^{H} \end{array} & \begin{array}{ccc} 0 & \cdots & 0\\ \vdots & 0 & \vdots \\ 0 & \cdots & 0 \end{array} & \begin{array}{ccc} -{\hat{e}}_{11}^{H} & \cdots & -{\hat{e}}_{1M}^{H}\\ \vdots & -{\hat{e}}_{j\mu }^{H} & \vdots \\ -{\hat{e}}_{I1}^{H} & \cdots & -{\hat{e}}_{IM}^{H} \end{array} & \begin{array}{ccc} {\hat{d}}_{11}^{H} & \cdots & {\hat{d}}_{1L}^{H}\\ \vdots & {\hat{d}}_{j\ell }^{H} & \vdots \\ {\hat{d}}_{I1}^{H} & \cdots & {\hat{d}}_{IL}^{H} \end{array}\\ \;\begin{array}{ccc} {\hat{f}}_{11}^{H} & \cdots & {\hat{f}}_{1I}^{H}\\ \vdots & {\hat{f}}_{\mu \tau }^{H} & \vdots \\ {\hat{f}}_{M1}^{H} & \cdots & {\hat{f}}_{MI}^{H} \end{array} & \begin{array}{ccc} 0 & \cdots & 0\\ \vdots & 0 & \vdots \\ 0 & \cdots & 0 \end{array} & \begin{array}{ccc} -k_{11}^{H} & \cdots & -k_{1M}^{H}\\ \vdots & -k_{\mu r}^{H} & \vdots \\ -k_{M1}^{H} & \cdots & -k_{MM}^{H} \end{array} & \begin{array}{ccc} {\hat{h}}_{11}^{H} & \cdots & {\hat{h}}_{1L}^{H}\\ \vdots & {\hat{h}}_{\mu \ell }^{H} & \vdots \\ {\hat{h}}_{M1}^{H} & \cdots & {\hat{h}}_{ML}^{H} \end{array}\\ \;\begin{array}{ccc} {\hat{x}}_{11}^{H} & \cdots & {\hat{x}}_{1I}^{H}\\ \vdots & {\hat{x}}_{\ell \tau }^{H} & \vdots \\ {\hat{x}}_{L1}^{H} & \cdots & {\hat{x}}_{LI}^{H} \end{array} & \;\begin{array}{ccc} 0 & \cdots & 0\\ \vdots & 0 & \vdots \\ 0 & \cdots & 0 \end{array} & \begin{array}{ccc} -{\hat{z}}_{11}^{H} & \cdots & -{\hat{z}}_{1M}^{H}\\ \vdots & -{\hat{z}}_{\ell \mu }^{H} & \vdots \\ -{\hat{z}}_{L1}^{H} & \cdots & -{\hat{z}}_{LM}^{H} \end{array} & \begin{array}{ccc} {\hat{y}}_{11}^{H} & \cdots & {\hat{y}}_{1L}^{H}\\ \vdots & {\hat{y}}_{\ell o}^{H} & \vdots \\ {\hat{y}}_{L1}^{H} & \cdots & {\hat{y}}_{LL}^{H} \end{array}\\ \;\begin{array}{ccc} {\hat{c}}_{11}^{P} & \cdots & {\hat{c}}_{1I}^{P}\\ \vdots & {\hat{c}}_{u\tau }^{P} & \vdots \\ {\hat{c}}_{Q1}^{P} & \cdots & {\hat{c}}_{QI}^{P} \end{array} & \;\begin{array}{ccc} 0 & \cdots & 0\\ \vdots & 0 & \vdots \\ 0 & \cdots & 0 \end{array} & \begin{array}{ccc} -{\hat{e}}_{11}^{P} & \cdots & -{\hat{e}}_{1M}^{P}\\ \vdots & -{\hat{e}}_{u\mu }^{P} & \vdots \\ -{\hat{e}}_{Q1}^{P} & \cdots & -{\hat{e}}_{QM}^{P} \end{array} & \begin{array}{ccc} {\hat{d}}_{11}^{P} & \cdots & {\hat{d}}_{1L}^{P}\\ \vdots & {\hat{d}}_{u\ell }^{P} & \vdots \\ {\hat{d}}_{Q1}^{P} & \cdots & {\hat{d}}_{QL}^{P} \end{array} \end{array}\right]\in {\mathbb{R}}^{\left(2I+2Q+L+M)\times (I+Q+L+M\right)} \end{array}$$

where HP, PP, HG, HM, HL and PG denote the host protein, pathogen protein, host gene, host miRNA, host lncRNA and pathogen gene, respectively; ${\hat{a}}_{in}^{H},\;{\hat{b}}_{iv}^{H}$ and ${\hat{a}}_{qn}^{P},\;{\hat{b}}_{qv}^{P}$ mentioned in (1) and (2) could be acquired in ${\hat{\theta }}_{i}^{HP}$ and ${\hat{\theta }}_{q}^{PP}$ by resolving the parameter estimation problems in (13) and (14) and pruning false positives by AIC method in (31) and (32), respectively; $\left\{{\hat{c}}_{j\tau }^{H},\;-{\hat{e}}_{j\mu }^{H},\;{\hat{d}}_{j\ell }^{H}\right\}$, $\left\{{\hat{f}}_{\mu \tau }^{H},\;-k_{\mu r}^{H},\;{\hat{h}}_{\mu \ell }^{H}\right\}$, $\left\{{\hat{x}}_{\ell \tau }^{H},\;-{\hat{z}}_{\ell \mu }^{H},\;{\hat{y}}_{\ell o}^{H}\right\}$, and $\left\{{\hat{c}}_{u\tau }^{P},-{\hat{e}}_{u\mu }^{P},{\hat{d}}_{u\ell }^{P}\right\}$ mentioned in (3)–(6) could be acquired in ${\hat{\theta }}_{j}^{HG},\;{\hat{\theta }}_{u}^{PG},\;{\hat{\theta }}_{\mu }^{HM}$ and ${\hat{\theta }}_{\ell }^{HL}$ by resolving the parameter estimation problems in (27)–(30) and pruning false positives by AIC method in (33)–(36) respectively. Note that since the regulations from pathogen proteins weren’t taken into consideration, the corresponding parameters in matrix A are padded with zeros.

Thereafter, we extract the core components of HPI-GEN by PNP approach, a principal network analysis method for dimensionality reduction based on network structure projection technique, that is, projecting matrix A to its principal singular vectors space. Accordingly, the combined network matrix A can be denoted by singular value decomposition form below,

(37) $$A=USV^{T}$$

where $U\in {\mathbb{R}}^{\left(2I+2Q+L+M)\times (I+Q+L+M\right)},\;V\in {\mathbb{R}}^{\left(I+Q+L+M)\times (I+Q+L+M\right)},\;\mathrm{and}\;S=diag(\sigma_{1},\ldots ,\sigma_{s},\ldots \sigma_{I+Q+L+M})$ is a diagonal matrix of $\sigma_{1},\ldots ,\sigma_{s},\ldots \sigma_{I+Q+L+M}$ which includes the I + Q + L + M singular values of the matrix A in descending order, i.e., $\sigma_{1}\geq \ldots \geq \sigma_{s}\geq \ldots \geq \sigma_{I+Q+L+M}\geq 0$.

$$S=\left[\begin{array}{cccccc} \sigma_{1} & 0 & \cdots & 0 & \cdots & 0\\ 0 & \sigma_{2} & \cdots & 0 & \cdots & 0\\ \vdots & \vdots & \ddots & \vdots & \ddots & \vdots \\ 0 & 0 & \cdots & \sigma_{s} & \cdots & 0\\ \vdots & \vdots & \ddots & \vdots & \ddots & \vdots \\ 0 & 0 & \cdots & 0 & \cdots & \sigma_{I+Q+L+M}\\ 0 & 0 & \cdots & 0 & \cdots & 0\\ \vdots & \vdots & \ddots & \vdots & \ddots & \vdots \\ 0 & 0 & \cdots & 0 & \cdots & 0 \end{array}\right]$$

Also, we define the expression fraction $E_{w}$ of the interaction ability of the proteins and the transcriptional regulatory ability of the genes, lncRNAs, miRNAs as the normalization of singular value form,

(38) $$E_{w}=\frac{\sigma_{w}^{2}}{\sum_{w=1}^{I+Q+L+M}\sigma_{w}^{2}}$$

According to the diminishing property of the singular values, under the basic premise of sustaining system energy from the whole network structure, we pick out the minimum X such that $\Sigma_{w=1}^{X}E_{w}\geq 0.85$, i.e., the vector space spanned by the orthonormal bases composed of the principal singular vectors corresponding to the top X singular values contains 85% energy of HPI-GEN structure. 

Afterwards, we define and apply the projection value of each node, including gene, miRNA, lncRNA, and protein in the real HPI-GEN to the vector space spanned by the orthonormal basis composed of the principal singular vectors corresponding to the top X singular values as below,

(39) $$\begin{array}{l} \mathrm{Pr}oj_{R}(A_{row,i},V^{T^{*}})={\left[\sum_{k=1}^{X}{\left(A_{row,i}v_{k}\right)}^{2}\right]}^{1/2}\\ \;\mathrm{Pr}oj_{C}(A_{col,j},U^{*})={\left[\sum_{k=1}^{X}{\left(A_{col,j}^{T}u_{k}\right)}^{2}\right]}^{1/2} \end{array}$$

where $A_{row,i}$ denotes the *i*th row vector of A for i = 1,…,2I+2Q+L+M; $A_{col,j}$ signifies *j*th column vector of A for j = 1,…,I+Q+L+M; $V^{T^{*}}$; and $U^{*}$ represent the vector spaces spanned by the orthonormal basis $\left\{v_{1},v_{2},\ldots ,v_{k}\right\}$ and $\left\{u_{1},u_{2},\ldots ,u_{k}\right\}$ for k=1,…,X, respectively; $\mathrm{Pr}oj_{R}(A_{row,i},V^{T^{*}})$ and $\mathrm{Pr}oj_{C}(A_{col,j},U^{*})$ indicate the projection value from $A_{row,i}$ and $A_{col,j}$ to $V^{T^{*}}$ and $U^{*}$ vector spaces, respectively.

While the projection value $\mathrm{Pr}oj_{R}(A_{row,i},V^{T^{*}})$ or $\mathrm{Pr}oj_{C}(A_{col,j},U^{*})$ in (39) approaches zero, it intimates that the corresponding node *i* or *j* isn’t the pivotal factor or independent to the core network extracted via the PNP procedure. Conversely, the larger projection value it gets, the greater the node contributes to the core network.

Eventually, we select the first 3000 components on the top of the list with higher energy in (39) as the core host-pathogen network from real HPI-GEN of HBV infection by ranking the projection value of each node and subsequently uploading those nodes into DAVID to obtain the KEGG pathways as shown in Table 3. Taking KEGG pathways as reference, we systematically identify and investigate the molecular pathogenic mechanism of HBV infection from the core HPI-GEN as shown in Figure 4 and select the significant biomarkers for the further novel drug discovery procedure. 

### 2.8. Deep Learning-Based Drug-Target Interaction Prediction

#### 2.8.1. Data Preparation

Following the identification of promising pathogenic biomarkers, a potent drug-target interaction (DTI) model to predict the interactions between the identified biomarkers and their corresponding molecule drugs is crucial for discovering favorable molecular compounds for the therapeutic treatment. Thus, we constructed a deep learning-based DTI model to collect viable interactions to identify available drugs for the selected biomarkers. Meanwhile, since most patients in need of attention and treatment are infected with chronic hepatitis, the highest priority was assigned to efficacious drugs with lower toxicity that would not cause irreparable harm to health.

First, we integrated databases from UniProt [53], DrugBank [54], ChEMBL [55], Pubchem [56], and BindingDB [57] to assemble applicable drug-target interactions. As feature descriptors are simple numerical vectors designed to delineate the complicated information of objects, they have been widely utilized to describe the genomic sequences and chemical properties of molecules such as the characteristics from 2D, 3D spectrum of structure, molecular weight, predictive values of LogP, etc. of late [58,59]. In view of this property, we employed both the off-the-shelf build-in molecular and protein descriptor functions of python package pyBioMed to transform the features from each drug and its target among the previously collected drug-target interactions into descriptor under python 2.7 environment, respectively. These features include molecular dockings such as amino acid composition and dipeptide composition. For more details about the descriptor transformation, readers could access the documents of pyBioMed. Thereafter, we united the descriptors of both drugs and their relevant targets into a matrix as the training dataset to train the DTI model. The vector $v_{drug-t\arg et}$ corresponding to the descriptor of each drug-target pair is given below:

(40) $$v_{drug-t\arg et}=[D,T]=[d_{1},d_{2},\cdots ,d_{I},t_{1},t_{2},\cdots t_{J}]$$

where $v_{drug-t\arg et}$ denotes the vector of the descriptor for each the drug-target pair comprising two parts. The former part D is the descriptor of the drug and $d_{i}$ represents the ith drug feature; the latter portion T is the descriptor of the target and $t_{j}$ indicates the jth target characteristic; *I* is the total number of drug features; *J* is the total number of target features. 

Before performing drug-target interaction prediction, to keep our DTI model from inferior performance arising from between-class imbalance and distracting variation, some data preprocessing procedures are requisite. The entire preprocessing procedure in our work encompasses down sampling, data partitioning, feature scaling, and dimensionality reduction.

- Down Sampling:

There are two categories of drug-target interaction in our samples: 16,000,000 samples for the unknown interactions (negative instances) and 60,000 samples for the known interactions (positive instances). Though deep learning methods scale well with the quantity of data and can often leverage extremely large datasets for good performance, imbalanced class distribution often causes the model to be overwhelmed by the large class and ignore the minority one. So as to mitigate the effect arising from class imbalance, we reduced the number of majority samples (negative instances) to 60,000 which will allow the model to learn from both classes equally.

- Data Partitioning:

After the down sampling procedure, we partition the data (the reconstructed matrix of drug-target pairs) into two sets, three-fourth for training and one-fourth for testing.

- Feature Scaling:

Since the variables of the features in each drug-target pair are measured at different scales, they do not contribute equally to the model fitting and might end up with creating a bias. To deal with this potential problem, a feature-wise scaling is usually implemented prior to model fitting. As powerful techniques of feature scaling, Min-max scaling (normalization) and Standardization are both widely used in data analysis. However, despite possessing the capability to shift and rescale the data into a limited range of values, normalization is sensitive to the outliers [60]; in contrast, standardization maintains useful information about outliers and makes the model less sensitive to them [61]. Thus, we apply Standardization on each feature and the corresponding mathematical formulation is shown as follows:

(41) $$\begin{array}{l} d_{i}^{*}=\frac{d_{i}-\mu_{i}}{\sigma_{i}},\;\forall i=1,\ldots ,I\\ t_{j}^{*}=\frac{t_{j}-\omega_{j}}{\delta_{j}},\;\forall j=1,\ldots ,J \end{array}$$

where $d_{i}$ and $d_{i}^{*}$ represent the ith feature of the drug before and after Standardization, respectively; $\mu_{i}$ and $\sigma_{i}$ signify the mean and standard deviation of the ith drug feature; $t_{j}$ and $t_{j}^{*}$ indicate the *j*th feature of the target before and after Standardization, respectively; $\omega_{j}$ and $\delta_{j}$ denote the mean and standard deviation of the ith target feature; *I* is the total number of features in the drug while *J* is that of the target.

- Dimensionality Reduction:

Since the high-dimensional patterns in samples might augment the number of neurons in the network and elevate the computational complexity for training, we adopted principal components analysis (PCA) to distill 692 features from original 1014 features of the samples. One could retain as much information of interested [62,63].

Note that it is illegal and useless to carry out data preprocessing to the testing set solely relying on the characteristics in testing data in that we would never get any information from the features of testing data until the training phase is over. Hence, aside from the aforementioned transformation performed on the training data sets, we also provide the identical transformation on the testing data during the process of feature scaling and dimensionality reduction. More exactly, we extracted the mean and standard deviation variables of each feature in the training data set to standardize the corresponding feature in the testing data. Likewise, the transformation matrix for executing PCA of the features in training data was concurrently used to reduce the dimension of the features in testing data.

So far, the training data for tuning the network parameters of the deep learning-based DTI model and the testing data for evaluating the model performance are ready. Nonetheless, if learned merely on the features from the training set, the hyperparameters would always choose the maximum possible model capacity, leading to overfitting [63]. As a result, we randomly split out one-tenth of the training data to estimate the generalization error during training, allowing for the hyperparameters to be updated in every epoch. In addition, Early Stopping method was also employed to specify an arbitrarily large number of training epochs and stop training once improving the model’s fit to the training data comes at the expense of increased generalization error. Eventually, through setting the optimizer as Adam [64] and learning rate = 0.001, we then trained our DTI model for 40 epochs with 100 samples in each mini-batch. 

#### 2.8.2. Parameters Tuning Process Based on Deep Learning Algorithm

In the network architecture, each layer can be simplified into a function below:

(42) $${\hat{h}}_{n}=\delta (w^{T}x_{n}+b)$$

where $x_{n}$ and ${\hat{h}}_{n}$ indicate the input and output vectors corresponding to the descriptor of the *n*th drug-target pair, respectively; δ denotes the activation function (ReLU for each hidden layer and Sigmoid for the output layer); and the vectors w and b are given as follows:

(43) $$w\;=\left[\begin{array}{c} w_{1}\\ w_{2}\\ \vdots \\ w_{e} \end{array}\right],\;b=\left[\begin{array}{c} b_{1}\\ b_{2}\\ \vdots \\ b_{e} \end{array}\right]$$

where the weights parameters $w_{1},w_{2},\ldots w_{e}$ in w and bias parameters $b_{1},b_{2},\ldots b_{e}$ in b are free variables that capture the model’s representation of the data in each layer and are learned from each sample. 

To evaluate the model performance, the predicted output is compared with the true label to compute a loss for the current set of model weights. Since drug-target interaction prediction issue is a binary classification problem, binary cross-entropy is chosen to calculate the loss of each iteration. The summation of the loss $L(y,\hat{y})$ for totally N samples is given by the expression as below:

(44) $$L(y,\hat{y})=-\frac{1}{N}\sum_{n=1}^{N}\left(y_{n}*\log({\hat{y}}_{n})+(1-y_{n})*\log(1-{\hat{y}}_{n})\right)=\frac{1}{N}\sum_{n=1}^{N}C^{n}(y_{n},{\hat{y}}_{n})$$

where $C^{n}(y_{n},{\hat{y}}_{n})$ is loss of the *n*th sample calculated by binary cross-entropy, in which $y_{n}$ is the *n*th actual label (1 or 0) and ${\hat{y}}_{n}$ is the *n*th predicted probability distribution.

During the learning process, the parameter set θ (46) in each layer should achieve the minimization of the objective function (47) to obtain the optimal network parameters $\theta^{*}$. Accordingly, backward propagation learning algorithm (48) is designed to update the network parameters of both weights ($w_{1},w_{2},\ldots ,w_{e}$) and bias ($b_{1},b_{2},\ldots ,b_{e}$).

(45) $$\theta =\left[\begin{array}{c} w_{1}\\ \vdots \\ w_{e}\\ b_{1}\\ \vdots \\ b_{e} \end{array}\right]$$

(46) $$\theta^{*}=\arg\underset{\theta }{\min}L(\theta )$$

(47) $$\theta^{i}=\theta^{i-1}-\eta \nabla L(\theta^{i-1})$$

where *i* is the *i*th iteration of the learning process; η is the learning rate (set as 0.001); and $\nabla L(\theta^{i-1})$ is the gradient of $L(\theta^{i-1})$ given as follows:

(48) $$\nabla L(\theta^{i-1})=\left[\begin{array}{c} \frac{\partial L(\theta^{i-1})}{\partial w_{1}}\\ \vdots \\ \frac{\partial L(\theta^{i-1})}{\partial w_{e}}\\ \frac{\partial L(\theta^{i-1})}{\partial b_{1}}\\ \vdots \\ \frac{\partial L(\theta^{i-1})}{\partial b_{e}} \end{array}\right]$$

Consequently, by back-propagating a corrective error signal through the network, weighted connections between neurons in the DTI model are iteratively adjusted and assessed on the basis of the drug-target pairs in the training and the validation sets. 

#### 2.8.3. Measurement of Prediction Quality 

Assessing a model is quite tricky. It isn’t reliable to evaluate model performance (training accuracy of the final epoch) merely based on one specific validation set, in that the accuracy obtained from one validation set can be very different to that from another. Thus, we took advantage of a general technique named 10-fold cross validation to avoid the possible bias. That is, the original samples for training is randomly partitioned into 10 equal size subsamples; one is retained as the validation data for testing the model, and the remaining 9 subsamples are used as training data. Through repeatedly training and validating the models 10 times with different partitions, we then compute an average score over the rounds to give an estimate of the model’s predictive performance. Afterwards, the model parameters with different validation errors were applied to the testing data for generating the final test accuracy and loss. 

Aside from that, to compare the effectiveness of our DTI model with that of other state-of-the-art ML-based approaches, we adopted AUC-ROC curve [65] to tell how good a specific model can distinguish two classes, that is, whether or not a drug interacts with a target. ROC represents the relationship between benefit (true positive rate) and cost (false positive rate), while AUC is a value in the range of 0 to 1 that indicates the degree of separability between classes. The higher AUC we obtain, the more accurate outcome we will get from the model. The formulas for AUC-ROC curve are shown as below:

(49) $$\mathrm{True}\;\mathrm{Positive}\;\mathrm{Rate}\;=\;\mathrm{Recall}/\mathrm{Sensitivity}\;=\;\frac{TP}{TP+FN}$$

(50) $$\mathrm{Specificity}\;=\frac{TN}{TN+FP}$$

(51) $$\mathrm{False}\;\mathrm{Positive}\;\mathrm{Rate}\;=\;1-\mathrm{Specificity}\;=\;\frac{FP}{TN+FP}$$

where True Positive (TP) means that the actual value is positive and is judged correctly; False Positive (FP) shows that the actual value is positive but is judged by mistake; True Negative (TN) indicates the actual value is negative and is judged accurately; False Negative (FN) represents the actual value is negative but is judged in error.

## 3. Results

### 3.1. Overview of Systems Medicine Discovery Procedure

Though a great advance has been made to design and evaluate medicine based on the application of diversified drug repositioning approaches, it still takes a large amount of time and vigor if without a potent avenue to select the promising biomarkers and drugs for the clinical trials. In this study, we proposed a systematic biology method containing two sections including potential biomarkers identification and novel drugs discovery. 

**Figure 1:**
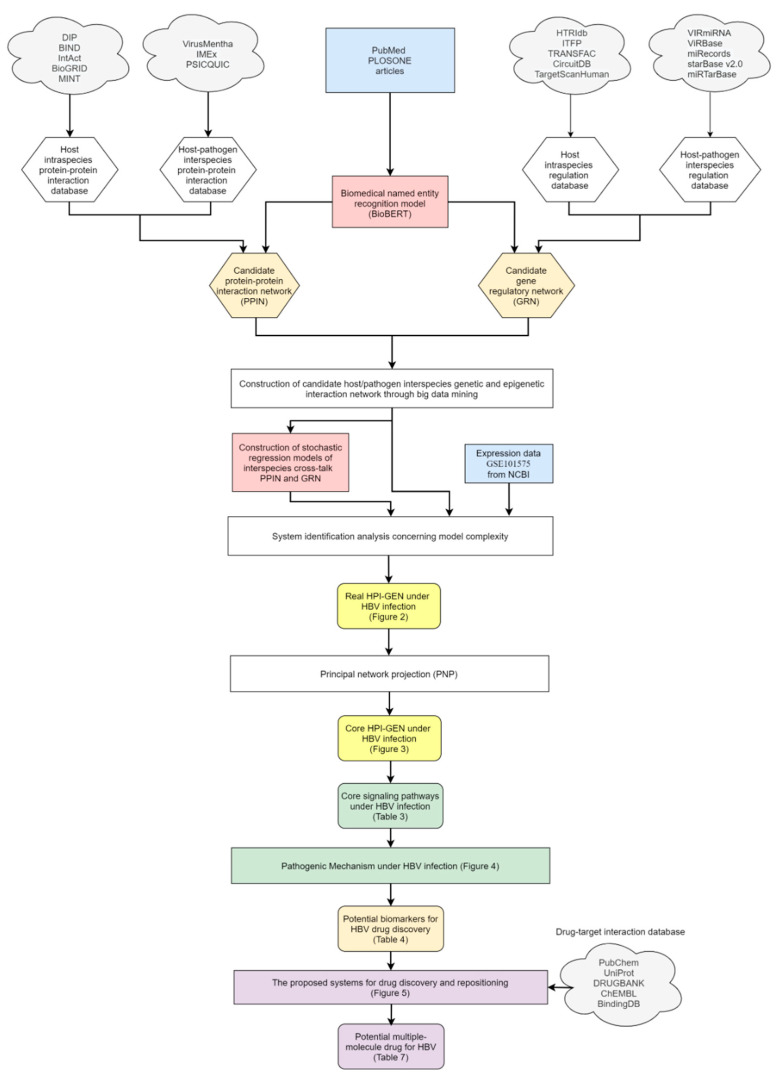
Flowchart of applying systems biology method to construct the candidate HPI-GEN, real HPI-GEN, core HPI-GEN, and core signaling pathways during the infection of HBV to identify the potential biomarker for later drug discovery repurposing. The grey blocks in the shape of a cloud indicate the online databases for constructing candidate HPI-GEN and later drug repositioning; the white diamond blocks are the corresponding data types of each database; the orange diamond blocks signify candidate protein-protein interaction network (PPIN) and gene regelation network (GRN); the blue blocks represent the input information including the raw RNA-seq data for system identification and the collected articles for BioBERT to augment the interspecies interactions and regulations in PPIN and GRN, respectively; the red blocks denote the models comprising BioBERT and stochastic regression models utilized in the systems biology approach; the white rectangle blocks indicate the systematic methods applied to constructing the candidate HPI-GEN and extracting core HPI-GEN; the purple blocks imply the drug discovery and repositioning procedure proposed afterwards; and the yellow rounded rectangular blocks are the real HPI-GEN and core HPI-GEN during the infection of HBV.

**Figure 2:**
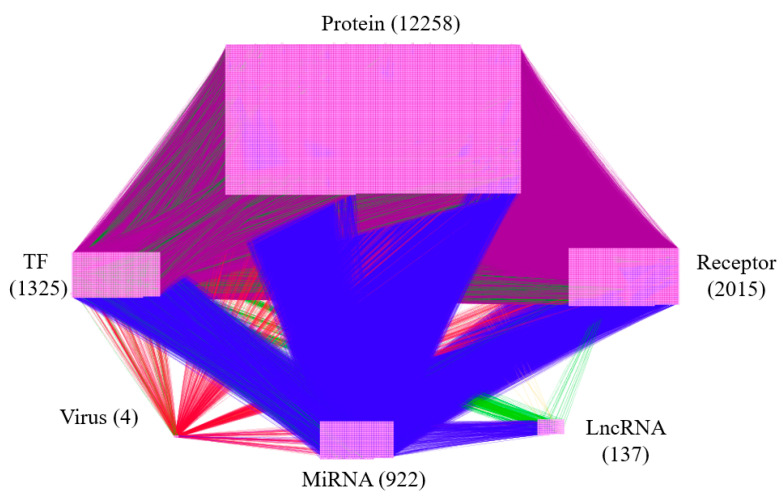
Real HPI-GEN between host and pathogen under infection of HBV. The real HPI-GEN describes the cross-talk interaction and regulation between host and pathogen after system identification analysis to prune false positives from candidate HPI-GEN. The purple lines denote PPIs; the green, yellow, blue, and red lines indicate the regulations by Transcriptional factors (TFs), lncRNAs, miRNAs, and Virus proteins respectively; and the total numbers of lncRNAs, miRNAs, proteins, receptors, Virus proteins, and TFs in the network are 137, 922, 12,258, 2015, 4, 1325, respectively.

**Figure 3:**
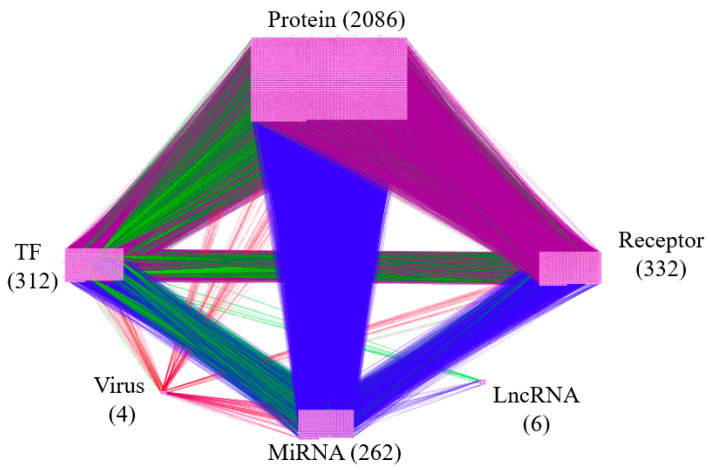
Core HPI-GEN under infection of HBV. The core HPI-GEN denotes the cross-talk interactions between host and pathogen after performing PNP to extract the top 3000 nodes from the real HPI-GEN. The purple lines denote PPIs; the green, yellow, blue, and red lines indicate the regulations by TFs, lncRNAs, miRNAs, and Virus proteins, respectively; and the total numbers of lncRNAs, miRNAs, proteins, receptors, Virus proteins, and TFs in the network are 6, 262, 2086, 332, 4, 312, respectively.

**Figure 4:**
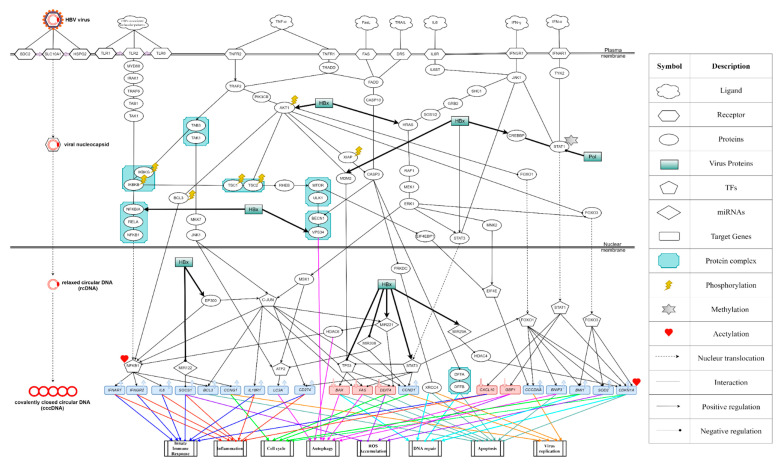
The core host/pathogen cross-talk signaling pathways during HBV infection. This is a network obtained from the core HPI-GEN by the annotation of KEGG (Kyoto Encyclopedia of Genes and Genomes) pathways, which represents an intracellular pathogenic mechanism during the invasion of host hepatocyte by HBV. The solid black lines indicate the regulation (with an arrowhead) and interaction between nodes. The solid lines in different colors except black represent the regulations of the corresponding cellular functions; among them, the lines with an arrowhead denote the activation of cellular functions while the lines with a circular head indicate the inhibition of cellular functions. Furthermore, for the nodes of downstream target genes, the blocks with an upward pointing arrow signify a rising expression; comparatively, the blocks with a downward pointing arrow signify a dropping expression under HBV infection.

The overall flowchart of the proposed systems biology procedure is shown in Figure 1. On one hand, we applied a systematic identification analysis procedure to the constructed candidate HPI-GEN, considering both accuracy and model complexity with the help of the information from the two-side real-time profile RNA-seq. Note that the total nodes and edges respectively given in Table 1 and Table 2 of the real HPI-GEN in Figure 2 abstracted from the candidate HPI-GEN has considerably shrunk in comparison with that of the candidate HPI-GEN, which indicates that the false-positives were eliminated during the identification process. Supported with the PNP approach, we then identified the core HPI-GEN in Figure 3 by selecting top-ranked 3000 nodes with significant projection values that could reflect 85% of the real HPI-GEN. The higher the projection value is, the stronger regulatory capacity it possesses in the host/pathogen cross-talk mechanism during HBV infection. In the meantime, given identified KEGG pathways derived through uploading nodes in core HPI-GEN into DAVID in Table 3, we selected the core signaling pathways so as to explore the underlying etiologic mechanism in HBV infection as shown in Figure 4, involving microenvironmental factors by surveying relevant papers and research. 

**Figure 5:**
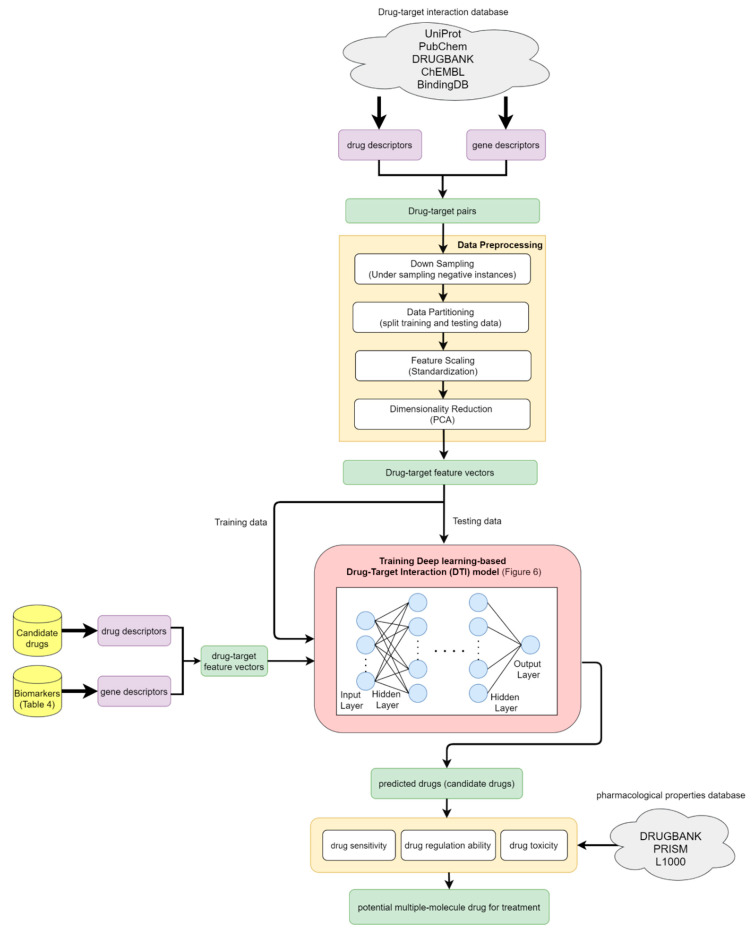
The flowchart of the proposed systems drug discovery and repositioning procedure. According to the potential biomarkers identified in Figure 4, we propose a systems drug discovery and repositioning method for figuring out promising multiple-molecule drug to alleviate HBV infection. First, we collected data in respect to drug-target interaction from various online databases. Then, we turned each drug and target into descriptors and assembled them into a matrix of drug-target pairs. To keep our model from poor performance, data preprocessing prior to training is indispensable. Accordingly, we adopted data preprocessing approaches including down sampling, data partitioning, feature scaling, and dimensionality reduction (For more details, readers can refer to Section 2.8.1 in Materials and Methods) based on the property of the data. After training the Deep learning-based drug-target interaction (DTI) model shown in Figure 6 with the constructed training data, we evaluated the model performance (as shown in Table 5 and Figure 7) and compared the model efficacy of DTI with that of other traditional machine learning (ML)-based methods (as shown in Figure 8). Since there is only a neuron in the output layer which delivers the probability of whether the relation (docking) between a drug and a target exists, the DTI model enabled us to stress the importance on specific interactions (with output score approaching 1) and to identify candidate drugs. Next, concerning the availability of the predicted drugs (candidate drugs) via general criteria for drug design specifications such as regulation ability, toxicity, and sensitivity obtained from databases, we further filtered promising drugs that modulate multiple molecular targets and possess the efficacy with lower dosages. After all, low-dose drugs are more attainable and cause less harm to patients. In this regard, a set of multi-target drugs with appropriate toxicity for clinical investigations were selected as a multiple-molecule drug to overcome HBV infection.

On the other hand, after determining the biomarkers from signaling pathways of the core HPI-GEN as shown in Table 4, we proposed a deep learning-based drug-target interaction (DTI) method to predict the potential interactions (dockings) between the identified biomarkers and their corresponding drugs for further candidate drugs discovery. The outcome of the DTI model is presented in a form of probability. The higher probability analyzed by the DTI model, the connection between the selected biomarker and the predicted drug is more likely to exist. However, given the enormous number of candidate drugs, the general drug specifications such as regulation ability, toxicity, and sensitivity from multiple databases were taken into consideration to reduce the search space and ensure the safety and efficacy in clinical trials. The pertinent flow-process diagram of the systems molecular drug design procedure is shown in Figure 5.

### 3.2. Extracting Core Signaling Pathways from Identified HPI-GEN and Core HPI-GEN in HBV Infection

For the purpose of analyzing the molecular mechanism under HBV infection, extracting core signaling pathways from candidate HPI-GEN is critical. The process to construct candidate HPI-GEN and identify real HPI-GEN as well as core HPI-GEN has been shown in the flowchart of Figure 1. 

Thereafter, we identified the real HPI-GEN via parameter identification concerning the cost of model complexity under the auspices of corresponding RNA-seq data to prune false positives from candidate HPI-GEN (refer to Section 2.4, Section 2.5, Section 2.6 for more details). The total numbers of the nodes of proteins, Transcription factors (TFs), miRNAs, lncRNAs, and Virus proteins as well as the edges of their interactions and regulations for the candidate HPI-GEN and identified real HPI-GEN are given in Table 1 and Table 2, respectively. In addition, through employing the network visualizing software Cytoscape, the network of real HPI-GEN under HBV infection is shown in Figure 2.

Since the immense complexity of real HPI-GEN limits the possibility to efficiently investigate the pathogenic mechanism under HBV infection, applying principal network projection (PNP) and subsequently abstracting the top 3000 nodes with significant projection values on the 85% pivotal network structures to distill core HPI-GEN are essential. Meanwhile, the results of core HPI-GEN under the infection of HBV is shown in Figure 3.

Moreover, we employed DAVID Bioinformatics Resources version 6.8 to analyze the enrichments of relative pathways and specific cellular functions of core HPI-GEN as shown in Table 3. 

Through the multifaceted assessment of the core HPI-GEN based on existing studies and the annotation of KEGG signaling pathways, we could attain the core signaling pathways associated with microenvironmental factors including cytokines, chemokines, etc., for the pathogenesis of HBV infection as represented in Figure 4. Then, on the basis of the investigating the consequence of the systematic pathogenic molecular mechanism, we could identify the potential biomarkers holding great promise for the development of therapeutic targets.

### 3.3. Analysis of Core Interspecies Cross-Talk Pathways to Investigate Host/Pathogen Offensive/Defensive Mechanism during HBV Infection

Based on the core host/pathogen cross-talk signaling pathways in Figure 4, several identified processes leading to dysfunction of antiviral machinery are investigated to afford a stepwise understanding of the host/pathogen offensive and defensive mechanism under HBV infection.

#### 3.3.1. Inception of HBV Infection 

Hepatitis B Virus (HBV) has a 3.2-kb circular and partially double-stranded DNA genome that contains four genes named S, C, P, and X genes coding for the envelope proteins, the core protein and a related protein termed precore protein, the viral DNA polymerase, and the multifunctional regulatory protein, respectively [66,67]. The journey of HBV virions starts from attaching to host hepatocytes through heparan sulfate proteoglycans (SDC2 and HSPG2) and promptly transfers to sodium acid cotransporter (SLC10A1), initiating the HBV life cycle. Once entering the cells, the viral nucleocapsid containing the partially double-stranded DNA, known as the relaxed circular DNA (rcDNA), will be released into the cytoplasm and transported into the nucleus. The plus strand of the rcDNA is then repaired and completed to generate the covalently closed circular (cccDNA), which later forms the HBV minichromosome and is transcribed into RNAs for viral replication [68].

#### 3.3.2. Liver Microenvironment and Immune Pathogenesis under HBV Infection

Inflammatory and innate immune responses impact the pathogenesis of numerous diseases and participate in the activation of common inflammatory mediators and regulatory pathways. On top of the Toll pathways that could be directly stimulated by the viral particles, in response to the foreign invasions, leukocytes, e.g., neutrophils and macrophages that phagocytose and kill pathogens, might simultaneously coordinate additional host responses by synthesizing a wide range of inflammatory mediators, such as TNF-α, IL-6, IFN-α, and IFN-γ [69]. These cytokines and chemokines provoke the secretion of antiviral effectors and a series of immunoregulatory mechanisms against HBV infection. In general, cellular and molecular events and interactions would effectively mitigate impending injury or infection during inflammatory responses [70]. However, along with the frequent recurrence of inflammation, once imbalances of the microenvironment occur, it might lead to abnormal epigenetic variations and even immune dysregulation [71], which consequently incurs viral relapse.

#### 3.3.3. Toll Pathway as the First Line of Defense against Infection

Toll-like receptors (TLRs) have long been recognized as the first line of antiviral immunity in that they initiate intracellular signaling pathways to induce antiviral mediators, such as interferons (IFNs) and other cytokines [72]. Upon the recognition of foreign invaders (HBV-associated molecular patterns), TLRs elicited phosphorylation of IKKβ (IKBKB) through several signaling transduction proteins, including MyD88, IRAK1, TRAF6, TAB1, and TAK1. IκBα (NFKBIA) is a downstream protein of IKKβ keeping NF-κB sequestered in an inactive state in cytoplasm [73]. When phosphorylated by IKKβ, IκBα dissociated from NF-κB, resulting in the nuclear translocation of NF-κB and the induction of its several targets, in which the *Interleukin 6* (*IL6*), the *interferon-alpha receptor 1* (*IFNAR1*), the *interferon-gamma receptor 2* (*IFNGR2*), and *BCL3* were recognized as shown in Figure 4. Among them, IL-6 is the pleiotropic cytokine encoded by *IL6* gene that catalyzes immune reaction and inflammatory responses [74,75]; IFNAR1 is the receptor that binds type I interferons and activates the JAK-STAT signaling pathway, along with MAPK, PI3K, and Akt signaling pathways associated with multiple cellular functions, such as inflammation and immune response regulation [76]; IFNGR2 is a receptor that binds to interferon-γ (IFN-γ), which is the immune interferon produced predominantly by natural killer (NK) and natural killer T (NKT) cells in response to viral infection [77]; and BCL3 is a protein that inhibits DNA damage-induced apoptosis and acts as a transcriptional coregulator of NF-κB [78,79]. 

In addition to stimulation of the downstream NF-κB pathway, the phosphorylated IKKβ also activated the pathway of mammalian target of rapamycin (mTOR) by triggering the disruption of TSC1/TSC2 complex. In general, TSC1 stabilizes TSC2 through direct binding, thereby preventing TSC2 from ubiquitination and degradation. Once IKKβ phosphorylates TSC1 incurring collapse of its connection with TSC2, it obliquely leads to the stabilization of RhebGTP, which mediates signaling to mTOR [80,81]. As for the substrates of mTOR, among all the identified downstream proteins in the core host/pathogen signaling pathways as shown in Figure 4, the translational repressor 4EBP1 (EIF4EBP1) stood out to be a pivotal constituent. It is well acknowledged that when the 4EBP1 protein is hyperphosphorylated by mTOR kinase, it can’t bind and hijack the eIF4E factor, i.e., a general initiator for translational regulation [82,83]. Concurrently, it is reported that the released eIF4E might form a complex with the 5′ stem-loop structure of the HBV genome, thus ensuring viral replication [84].

#### 3.3.4. TNF-α-Stimulated Signaling Pathways

TNF-α is a multifunctional cytokine that plays a principal role in cell proliferation, apoptosis, inflammation, and immune response. From the core host/pathogen cross-talk signaling pathways in Figure 4, as soon as TNF-α bound to TNF receptors (TNFRs), it contributed to the phosphorylation of Akt via both TNFR2/TRAF2/PIK3CB and TNFR1/TRADD/TRAF2/PIK3CB signaling transductions. Akt is an effector of Class I PI3K triggering multiple downstream pathways and has been shown to be a critical mediator of cell proliferation and survival in a variety of cell types [85]. In Figure 4, a few pathways of Akt were incorporated into the core host/pathogen cross-talk network. First of all, the activated Akt inhibited the induction of autophagy through transactivating mTOR, i.e., impeding GTPase-activating protein complex TSC1-TSC2 as previously described. It is worth noting that TSC2 rather than TSC1 was directly phosphorylated by Akt, which destabilized TSC2 and disrupted its affinity with TSC1 [86]. Secondly, Akt also enhanced MDM2-mediated ubiquitination leading to p53 (TP53) degradation [87] and catalyzed the nuclear localization and stabilization of BCL3 by phosphorylation [79], where BCL3 is the aforementioned transcription coregulator having been proven to interact with NF-κB so as to facilitate its transcriptional ability. Moreover, it even caused nuclear exclusion and cellular dysfunctions of FoxO proteins through phosphorylation and repression [88]. Therefore, as the identified target genes of FoxO proteins in the core host/pathogen cross-talk pathways, BNIP3, BMI1, SOD2, and cyclin-dependent kinase inhibitor 1 (CDKN1A) encoding p21, might probably be down-regulated. Within, BNIP3 is a pro-apoptotic factor that induces cell death through interacting with anti-apoptotic proteins [89]; BMI1 is a critical epigenetic repressor that functions through chromatin remodeling and plays a central role in DNA damage repair [90,91]; and SOD2 is the antioxidant defense enzyme that participates in apoptotic signaling and oxidative stress regulation [92]. However, it is well documented that FoxO1/FoxO3 could rapidly relocalize into the nucleus in response to STAT3 activation by IL-6, and the accumulation of FoxO1/FoxO3 in nuclei coincided with elevated expression of CDKN1A and other genes [93]. This phenomenon could interpret the high expression of the target genes BNIP3, BMI1, SOD2, and CDKN1A, as well as the promotion of viral replication, since activator FOXO1 has also been found to bind to HBV DNA and activate its transcription [94].

Aside from the activation of PI3K/Akt pathway, in response to cytokine signals, TRAF2 also recruited TAK1/TAB3 complex and IKK complex, which is composed of IKKα, IKKβ, and IKKγ (IKBKG), bringing about the autophosphorylation-dependent activation of TAK1 [95] and the activation of its substrates, such as IKKβ and MKK7. MKK7 then upregulated the transcriptional activity of c-JUN and ATF2 via activation of the JNK1. c-Jun is an AP-1 family transcription factor involved in the regulation of cell death and survival [96] as well as inflammation and innate immune response [97]. In Figure 4, the induction of c-Jun is discovered to be associated with the overexpression of the proinflammatory cytokine receptor IL18R1 essential for IL18 mediated signal transduction [98], the ligand PD-L1 (CD274) regulating the cellular immune responses to prevent inflammation [99], and the major cyclins CCND1 controlling the progression of a cell through the G1 phase by conjugating CDK4/6 enzymes required for the synthesis of cell cycle [100]. Besides, c-Jun not only modulated the expression of BCL3 by serving as either the coactivator of NF-κB or the activator of BCL3 gene to reinforce cell survival [101,102], but also induced miR-221 to interfere with the regulation ability of the histone deacetylase 6 (HDAC6), which has been reported to both suppress NF-κB activation and control autophagosome maturation essential for ubiquitin-selective quality-control autophagy [103]. Apart from that, c-Jun could even impact the modulation of *Fas* expression, which sparked off the loss of Fas-ligand (FasL)-mediated apoptosis by means of down-regulating the activity of p53.

#### 3.3.5. TAK-STAT Signaling Pathways

The Janus kinase (JAK)-signal transducer and activator of transcription (STAT) signaling pathway is a chain of interactions between proteins involved in various physiological processes, including immune function, cell growth, and cell death [104]. The activation of STAT3 was mainly provoked by JAK1 while JAK1 was brought in the proximity of receptors including the Glycoprotein 130 (IL6ST) and the gamma interferon receptor 1 (IFNGR1), which receive the signal from IL-6 and IFN-γ, respectively. However, the activation of STAT1 primarily stemmed from the phosphorylation of TYK2, a member of JAK family that was previously described by other research groups [105], by the alpha interferon receptor 1 (IFNAR1) residing in IFN-α stimulation. In spite of forming homodimers or heterodimers and translocating to the nucleus where they regulated the transcription of target genes, STAT3 and STAT1 played different even in part opposite roles in the process of pathogen infection. STAT3 constitutive activity is essential for cell survival and the extent of inflammation. Among the identified STAT3 targets in the host/pathogen cross-talk signaling pathway in Figure 4, an anti-inflammatory and anti-apoptosis factor referred to as *PD-L1* and the major cyclin *CCND1* involved in proliferation and cell cycle progression as mentioned previously were included. Additionally, STAT3 could also inhibit apoptosis by suppressing *Fas* transcription and p53 expression.

In contrast, STAT1 promoted apoptosis by inducing the expression of *CXCL10* and strengthened the antiviral ability through modulating the expression of *GBP1.* Amongst these two downstream targets, CXCL10 is a ligand for the receptor CXCR3 involved in chemotaxis, induction of apoptosis, and regulation of cell growth associated with a variety of human diseases including Hepatitis C [106] and Endotheliitis [107]. GBP1 is a binding protein which has been shown to provide broad host protection against different pathogen classes through expediting oxidative killing and delivering antimicrobial peptides to autophagolysosomes [108]. Furthermore, STAT1 also negatively regulated the cell cycle though inducing the expression of the CDK inhibitors *CDKN1A*, a target of transcription factors FoxOs as stated previously. 

Albeit STAT3 was directly activated by JAK1, it could also be indirectly triggered through the cytoplasmic signaling cascade of SHC/ERK pathway composed of signaling proteins, i.e., SHC1, GRB2, and SOS1, and the classical MAPK/ERK pathway comprised of signaling proteins, i.e., HRAS, RAF1, MEK1, and ERK1. Except inducing the activation of STAT3, MAPK/ERK pathway also contributed to the MSK1-mediated phosphorylation of c-Jun and ATF2, the inhibition of FoxO3 nuclear translocation, and most importantly, the regulation of eIF4E activity having been elucidated chiefly via phosphorylation on serine 209 of eIF4E by other research groups [83,109], which ultimately proliferated viral replication. More exactly, this was carried out by mitogen-activated kinase2 (MNK2), the downstream kinase of ERK1.

#### 3.3.6. TNFs-Induced Apoptotic Pathways

As a component of the cell response to viral infection, apoptosis mediated by the sequential activation of caspases is indispensable for aborting the production and release of progeny virus. While diverse signaling pathways inducing apoptosis have been found, caspase-mediated proteolysis of downstream substrates is still a critical element of the execution pathway common to all forms of apoptosis [110]. And among all the avenues conducive to apoptosis progression, the pathways contributing to the modulation of caspase-3 (CASP3), a frequently activated death protease that catalyzes the specific cleavage of many integral cellular proteins, occupied a key position and a majority of them began with the activation of FADD in Figure 4.

Induction of FADD, the upstream activating protein of caspase-10 (CASP10), was mediated by not only the bifurcated TNFR1-TRADD signaling cascades but also the interaction with the FasL receptor (FAS) and the TNF-related apoptosis-inducing ligand receptor (DR5). Upon stimulation, FADD in turn recruited the initiator caspase caspase-10 to form the death-inducing signaling complex, which leads to activation of a caspase cascade and eventual cell-death. Having been shown to activate the executioner caspase-3 through direct processing, caspase-10 is described to be functional-independent to caspase-8 (CASP8) in initiating Fas- and tumor necrosis factor-related ligand-mediated apoptosis [111,112]. Based on the cleavage potential of caspase-3 associated with the dismantling of the cell and the formation of apoptotic bodies, the identified critical targets including Beclin-1 (BECN1), DNA-PK (PRKDC), and the DNA fragmentation factor subunit alpha (DFFA) were specifically cleaved by caspase-3 with subsequent loss of its original capability. Among them, BECN1 is the component of the phosphatidylinositol-3-kinase (PI3K) complex that induces the appearance of autophagosome [113]; PRKDC is the nuclear serine/threonine protein kinase that takes part in degraded DNA repair [114]; and DFFA is a subunit of DFF. Further, according to previous study, whilst DFFA is cleaved, the DNA fragmentation factor subunit beta (DFFB) would dissociate from the cleaved fragments of DFFA and trigger both DNA fragmentation and chromatin condensation during apoptosis [115]. 

Additionally, it is worthwhile to note that Akt, including AKT1 and AKT2, has been reported to interact with X-linked inhibitor of apoptosis protein (XIAP) and protect it from ubiquitination and degradation [116]. Once phosphorylated by Akt, the XIAP would exploit its second baculovirus IAP repeat domain (BIR2) to inhibit the apoptotic executioner caspase-3 [117]. We believe that this identified episode carries a foreshadowing for the succeeding virus-induced malfunctions of programmed cell death.

#### 3.3.7. Virus Proteins-Induced Pathogenic Mechanism 

Proteins encoded by the viral genomes, especially HBx, a 154 amino acid gene product of the X gene, have emerged as promiscuous transactivators activating the transcription of host genes by interacting directly with nuclear transcription factors or by obliquely activating various signal transduction pathways in the cytoplasm and have been proven to be potent epigenetic modifying factors in hepatocytes. 

HBx regulated the expression of *BCL3* to subvert apoptotic signaling pathways via both synergistically cooperating with p300 (EP300) to enhance c-Jun transcriptional activity and activating NF-κB by either directly interacting with NF-κB for augmenting its binding ability to its target DNA sequence or promoting phosphorylation of IκB as shown in Figure 4. In addition, apoptosis inhibition could also be induced by dysfunction of caspase-3 through enhancing the ability of Akt, an upstream kinase of caspase-3 as stated earlier, by HBx. Once caspase-3 lost its function, in response to signal from damaged DNA, DNA-PKs, the substrate of caspase-3, would act in concert with XRCC4 and a number of tightly coupled proteins to repair DNA. Further, it was suggested that the intense activity or high expression of XRCC4 might be beneficial to the promotion of HBV cccRNA formation and thereby facilitate the viral replication [118]. Since Hepatitis B Virus supremely requires components from the host cell to complete its replication cycles, merely one developed avenue to hijack host signaling pathways can’t meet its demand. In addition to the significant translation initiation factor eIF4E induced by HBx through Akt/mTOR pathway as just mentioned, eIF4E was activated via the MAPK/ERK pathway as HBx could also trigger Ras and its downstream signaling pathways. Hence, forming a node of convergence of the PI3K/Akt/mTOR and Ras/Raf/MAPK signaling pathways, the regulation of eIF4E is clearly consequential for HBx to promote viral replication. Additionally, overexpression of cyclin G1 (CCNG1) resulted from the loss of microRNA-122 (a liver-specific miRNA) induced by HBx could also augment virus proliferation. It was reported that CCNG1 specifically interacts with p53 and blocks its specific binding to HBV enhancer elements, which simultaneously abrogates p53-mediated inhibition of HBV transcription and releases cell proliferation from G1/S arrest [119].

p53 acts as a sequence-specific DNA binding protein that activates the transcription of substantial target genes associating with the regulation of cell cycle progression, apoptosis, DNA repair, and senescence. In Figure 4, HBx interacted with p53 both directly and indirectly to inhibit its DNA consensus sequence binding and transcriptional activity, which has been reported to efficiently block p53-mediated cellular functions [120,121]. Apart from that, HBx also inhibited p53 by conjugating c-Jun and STAT3 as well as keeping MDM2, the principal cellular antagonist of p53 acting to limit the p53 growth-suppressive function [122], from ubiquitin-directed self-degradation. 

As for the targets of p53, apoptosis-associated proteins, such as the proapoptotic protein Bax, which could permeabilize mitochondria and engage the apoptotic program giving rise to activation of caspase-3 [123,124], the apoptosis antigen Fas triggering the Fas signaling pathway as mentioned earlier, and the DDIT4 negatively regulating cell growth, proliferation, and survival via inhibition of the activity of mTOR [125] were identified in our core host/pathogen cross-talk signaling pathways in Figure 4. Further, having been reported to participate in the generation of ROS and p53-dependent DNA damage [126], DDIT4 was negative-regulated by the HBx-induced upregulation of microRNA-221 to promote aberrant proliferation. Such significant inverse correlation between DDIT4 mRNA levels and miR-221 expression has been observed in a mouse model of liver cancer as well [127]. Besides, the direct suppression of histone deacetylases 6 (HDAC6) by HBx-induced miR-221 would result in autophagosome maturation failure; nevertheless, HBx yet also bound to Vps34 (PI3KC3) to enhance its activity, inducing the autophagic response crucial for the formation of autophagosomes as well as the intensification of viral replication.

STAT1 and STAT3 are both critical elements in viral replication, despite acting as distinct roles from each other. Functional characterization of miR-338-3p indicated that miR-338-3p inhibited cell proliferation by inducing cell cycle arrest at the G1/S phase. The identified downregulation of miR-338-3p and tyrosine phosphorylation of STAT3 triggered by HBx might cause *CCND1* overexpression beneficial to virus cell cycle progression [128]. In addition, forced low expression of miR-338 by HBx also directly led to the increased level of autophagy. This process was mainly through the up-regulation of LC3 by the released transcription factor ATF2, which has been identified as a direct target of miR-338 [129]. Furthermore, instead of FoxO3, HBx appeared to assist FoxO1 in counteracting the reduction of its nuclear relocalization arising from Akt phosphorylation by inducing STAT3 activation, which is conducive to the transcription of cccDNA as previously described. In contrast, since interferons (IFNs) activated STATs via phosphorylation [130], once recruited by HBx, histone acetyltransferase (HAT) CBP (CEBBP) might dynamically regulate STAT1 acetylation to counteract IFN-catalyzed STAT1 phosphorylation, nuclear translocation, DNA binding, and target gene regulation. On the other hand, STAT1 nuclear translocation induced by IFN-α might also be deprived by HBV polymerase [131]. Previous studies demonstrated that miR-122 regulates type I IFN expression through both down-regulating SOCS1 and SOCS3 to enhance interferon-mediated suppression of HBV [132,133]. Nonetheless, it appears that merely the regulation of SOCS1 by miR-122 was identified in Figure 4. Finally, as a target gene of FoxOs and STAT1, *CDKN1A* overexpression was mediated by HBx-induced upregulation of miR-29a. Serving as a direct upstream of HDAC4, miR-29a could thereby alleviate the deacetylation of *CDKN1A* through HDAC4 inhibition. 

### 3.4. Drugs Discovery and Repositioning Based on Selected Biomarkers for HBV

Through reviewing the cellular dysfunctions triggered by the core host/pathogen cross-talk interspecies signaling pathways under HBV infection shown in Figure 4, we selected the significant biomarkers including AKT1, NFKBIA, EIF4EBP1, HDAC6, STAT1, STAT3, and TP53 as drug targets shown in Table 4. However, there is still a long way to the clinical trials on account of the intrinsic difficulties for drug design and development. To this end, we proposed a systematic drug discovery and repositioning approach to uncover the multiple-molecule drug for therapeutic treatment based on the deep learning and data mining technique as shown in Figure 5. 

#### 3.4.1. Deep Learning-Based Drug-Target Interaction Prediction Model

**Figure 6:**
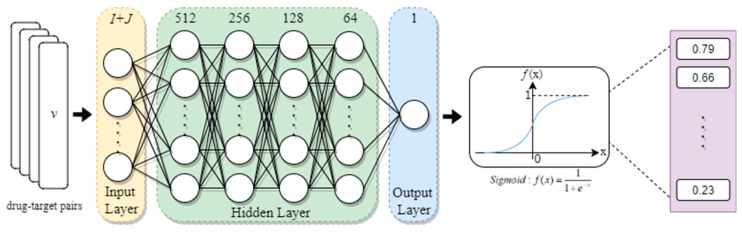
Configuration of the deep learning-based DTI model. v is the vector of each drug-target pair for the input layer; I is the total number of drug features; and J is the total number of target features.

To effectively predict whether the identified targets interact with some existing drugs, we provide a deep learning-based DTI model as shown in Figure 6. It is a fully-connected neural network containing an input layer and five layers with weights; four hidden layers (512, 256, 128, and 64 neurons in each layer) and an output layer (1 neuron). The intermediate hidden layers are applied with rectified linear unit (ReLU) activation function that thresholds negative signals to 0 and passes through positive signal. This type of activation function allows faster learning in comparison with alternatives, e.g., sigmoid or tanh unit [134]. In contrast, the output of the last layer is fed to a sigmoid function which produces a likelihood probability (0,1) score of the corresponding predicted docking where a higher value symbolizes a more reliable drug-target interaction. In addition, to further reducing overfitting, we incorporated a dropout layer [135] with a probability of 0.5 following the output of each hidden layer.

**Figure 7:**
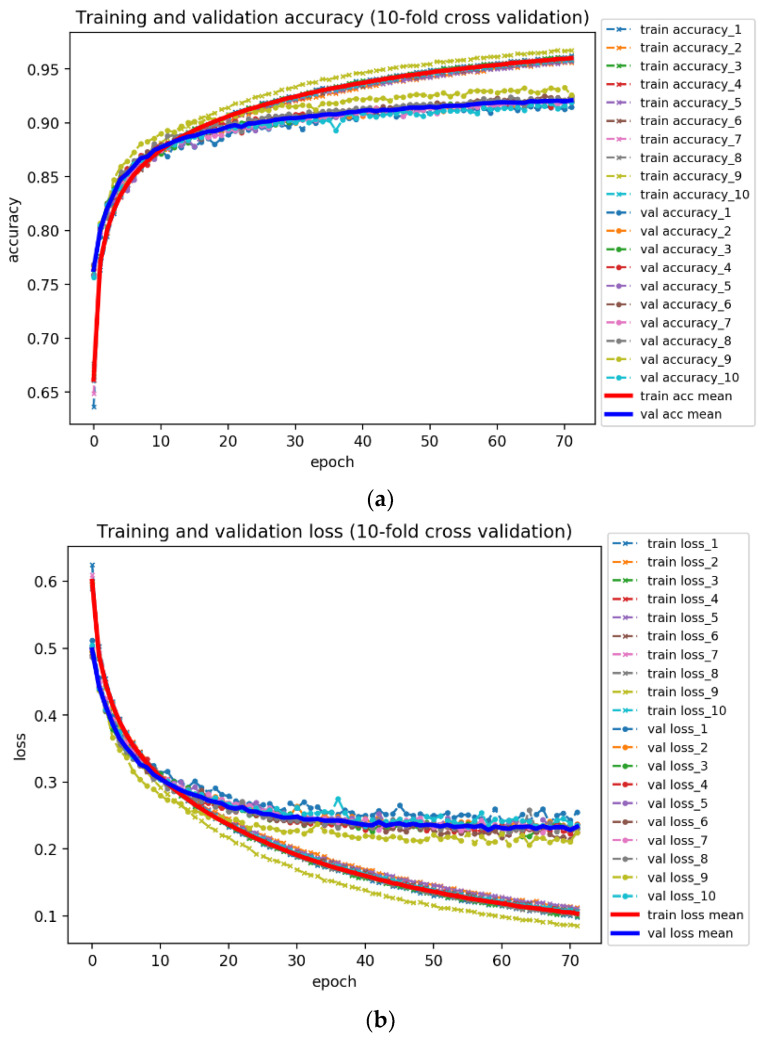
Training and Validation Learning curves (10-fold cross validation). “-x-” lines in different colors denote the training accuracy and loss, while “-o-” lines in different colors represent the validation accuracy and loss in (**a**,**b**), respectively. The bold lines in red and blue indicate the model’s average loss and accuracy of training and validation in (**a**,**b**), respectively. Moreover, since stopping the training of the neural network early before it has overfitted the training dataset can reduce overfitting and improve the generalization of the DTI model, Early Stopping approach is additionally employed to automatically stop the learning process at the epoch of 70.

For avoiding a poor performance due to assessing the model with a data set unfairly sampled (outliers or other anomalies are presented in one set), we introduced 10-fold cross validation measure to authenticate the learning effectiveness as shown in Table 5 and the corresponding learning curves that plot the learning performance of DTI model over experience and time are also presented in Figure 7. Note that since the model has been diagnosed well-fit and continuous training will likely lead to an overfit, it automatically stops learning at the epoch of 70. Subsequently, via calculating the loss and accuracy of each DTI model yields on the test set, we received an average accuracy of 92.6 (%) with the standard deviation of 0.294 (%). 

**Figure 8:**
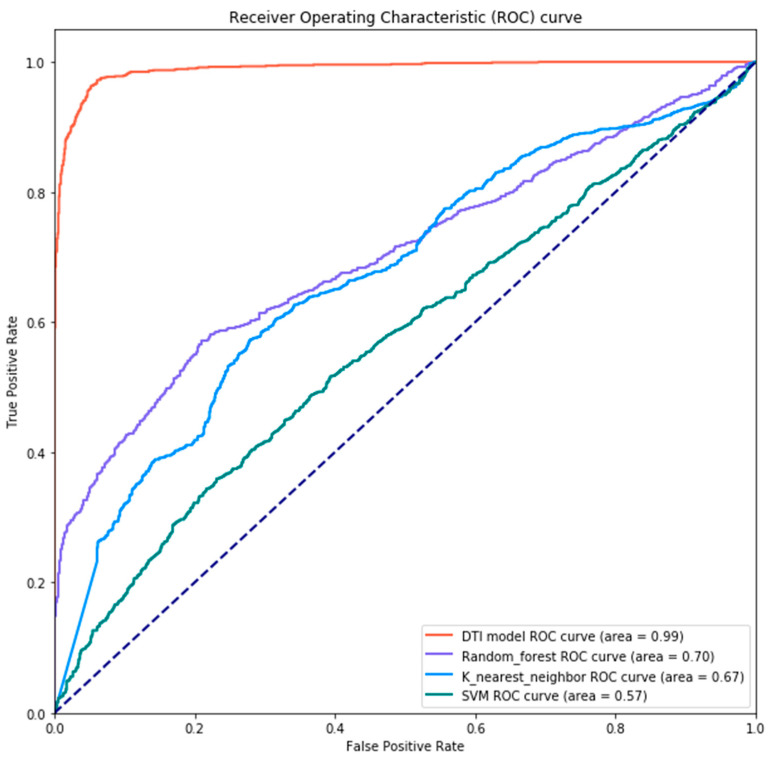
The Receiver Operating Characteristic (ROC) curve of models based on different methods. The accuracy of each method is calculated by the area under the corresponding ROC curve (AUC) (Readers can refer to Section 2.8.3 for further illustration), where the DTI model could achieve an accuracy of 0.99, which is higher than that of Random Forest (RF) (accuracy of 0.70), Nearest Neighbor (accuracy of 0.67), and Support Vector Machine (SVM) (accuracy of 0.57).

Moreover, with the increasement of experimental data, numerous machine learning approaches such as Random Forest (RF) [136], Support Vector Machine (SVM) [137], Nearest Neighbor [138], etc. have been applied to predict drug-target interactions. Therefore, to evaluate the model performance, we contrast our DTI model with a few of them, which are RF, Nearest Neighbor, and SVM. By adopting the visualization tool, Receiver Operating characteristic (ROC), we can clearly find that DTI outperforms other traditional binary classifiers as presented in Figure 8.

#### 3.4.2. Systems Discovery and Design of the Multiple-Molecule Drug for HBV Infection

On the basis of the pre-trained DTI model, we could evaluate the docking ability between the identified biomarkers and the available drugs. The larger predicted result we obtain, the higher probability that the specific drug-target pair will have interaction. Namely, the candidate drugs would be sifted out owing to holding higher probability (with output score approaching 1) to interact with the identified biomarkers. However, the exploration of promising drugs from a large quantity of the candidates (predicted drugs) is still arduous. Accordingly, in order to narrow down the scope of the possible options and improve the reliability of the ultimate selected drug combination, general criteria for drug design specifications including regulation ability, toxicity, and sensitivity were applied to further filter out unavailable or inappropriate medications. 

Under the criteria of the pharmacological properties, the regulation ability value for each drug was extracted from the LINCS L1000 level 5 dataset consisting of results from 978 genes of 75 cell lines treated with 19,811 small molecular compounds. By referring to LINCS L1000, we can examine whether a specific gene was up-regulated (positive values) or down-regulated (negative values) after being treated with a small molecular compound. Screening through a positive-regulation of NFKBIA, EIF4EBP1, HDAC6, STAT1, and TP53 and the negative-regulation of AKT1 and STAT3, the remaining drugs with agreeable regulation ability for the treatment of HBV-infected patients are shown in Table 6. 

Subsequently, to further narrow down the possibilities for drug combinations, the drug toxicity specification (LD50) recorded in DrugBank as well as the drug sensitivity specification (EC50) obtained from the PRISM dataset, which is a dataset comprising sensitivity values of 4518 drugs tested across 578 human cell lines, were also adopted. Being the numeric index of lethality, median lethal dose (LD50) plays a pivotal role in drug safety evaluation [139]. The drug with lower LD50 possesses higher toxicity, and thus it is more detrimental. On the other hand, half maximal effective concentration (EC50) is used as a measurement of concentration, which is often adopted to evaluate the suitability and the efficacy of drugs, where lower EC50 indicates the stronger inducibility, i.e., higher sensitivity. Based on these concepts, the drug combination comprising Sorafenib, Nutlin-3, and Tenofovir as shown in Table 7 was then designed as the multiple-molecule drug to rejuvenate the dysfunctions in the pathogenic mechanism under HBV infection with minor collateral tissue damage.

Among them, Sorafenib is a multikinase inhibitor drug having been approved for several tumor types, including Hepatocellular Carcinoma (HCC) [140]. Although prominent effects of Sorafenib for apoptosis induction were recognized, several limitations have been indicated by numerous medical studies upon applying Sorafenib to various cancers as a single agent. Therefore, the use of Sorafenib in combination with other targeted agents appears to be the main strategy for combatting drug resistance and has been widely explored with promising results [141]. Falling under the category of small molecule inhibitor, Nutlin-3 is conducive to the restoration of p53 pro-apoptotic ability, leading to the activation of cell cycle arrest and apoptosis pathways [142]. Though it is a novel antitumor compound, which hasn’t been clinically approved, the synergistic effect of Nutlin-3 combined with other agents contributes to diminishing of the dose required in monotherapy and decreasing the occurrence of adverse drug events [143,144]. Being a potent nucleotide analogue reverse-transcriptase inhibitor, Tenofovir is highly active against human immunodeficiency virus (HIV). In spite of its clinical use associated with the risk of kidney injury [145], it has been shown highly effective to patients who have never undergone an antiretroviral therapy [146]. Owing to its low toxicity, trifle side effects, and subtle drug resistance compared to other antivirals, Tenofovir is thereby frequently used as a member of combined drugs against HBV to ensure stronger antiviral activity and a more favorable safety profile [147,148]. It is worth noting that while therapy with Tenofovir is commonly used to treat Hepatitis B, life-long therapy is needed, which often increases the risk of relapse. In this respect, the combination of other viable drugs with Tenofovir may have a chance to improve the therapeutic effect and shorten the course of therapy.

Taken together, by administering the proposed multiple-molecule drug, an up-regulation of NFKBIA, EIF4EBP1, HDAC6, STAT1, and TP53 accompanied by the down-regulation of AKT1 and STAT3 can plausibly be attained, yielding encouraging results for the treatment of HBV-infected patients.

## 4. Discussion

Traditional drugs commonly used for treatment in patients afflicted with HBV are interferon and nucleoside analogues. However, the low therapeutic response of patients to interferon and the inevitable development of drug resistance to nucleoside analogues render the clinical treatment challenging [149]. In addition, serious toxic accumulation of the nucleoside analogues during long-term therapy for HBV infection is nonnegligible as well. Therefore, great efforts have been placed into seeking highly specific ligands affecting single target to alleviate HBV replication and to treat HBV-related complications. Yet, in view of the prohibitive development costs and timeline of a newly designed drug as well as the frequent failure of monotherapy due to drug resistance, growing interest has centered on discovering effective combinations of drugs for HBV treatment.

In this study, we develop a medicine discovery and repositioning procedure in terms of system identification approaches via two-side RNA-seq data and deep learning-based framework considering drug design specifications to find a possible drug combination for the treatment of HBV infection. Through investigation of core HPI-GEN annotated with KEGG pathways, the core signaling pathways responsible for the abnormally regulated cellular functions were extracted and unified in Figure 4. However, distinct cooperation between these pathways sparks diversified implication. Consequently, further discussion of the integrated effect is conducive to the selection of potential biomarkers in relation to the remedy of HBV infection.

### 4.1. Apoptosis in HBV Infection

As a fundamental and highly regulated biological process, apoptosis is a form of programmed cell death that participates in cell resistance to viral infection. Although mounting evidence shows that HBx is uniquely endowed with the capability to regulate apoptosis, its dual role as an apoptotic mediator raises the difficulty to elucidate the pathological mechanism [150].

Caspase-3 is the typical hallmark of apoptosis [110]. Numerous studies have shown that HBx abrogates the activity of caspase-3 [151,152,153], resulting in resistance to assorted apoptotic stimuli. In Figure 4, HBx-mediated inhibition activity of caspase-3 was chiefly through both activating XIAP, the inhibitor of caspase-3, and directly or obliquely regulating transcription factors such as NF-κB and p53 to affect the expression of their downstream anti-apoptotic target, e.g., *BCL3* and *Bax*. Further, even if previous researches did not specifically indicate the paramount downstream anti-apoptotic genes of NF-κB [154,155], BCL3, which hampers DNA damage-induced apoptosis and serves as a transcriptional coregulator of NF-κB [78,79], counted for NF-κB -mediated anti-apoptosis a lot in the pathogenic mechanism. Furthermore, inhibition of caspase-3 not only abrogated apoptotic killing triggered by the pathways in response to TNF-α, FasL, and TRAIL stimulation, but also contributed to the activation of autophagy, leading towards alleviated apoptosis coming from caspase-3 loss of its function to cleave Beclin-1.

Asides from that, *Fas*, indispensable for the FasL-induced pathway that gives rise to the activation of caspase-3, was downregulated during HBV infection. Negative-expression of *Fas* might impede the sustainable anti-viral response of death ligand stimulus-specific apoptotic pathway. In contrast, as a target gene relevant to execution and completion of apoptosis, the overexpressed *CDKN1A* triggered by HBx-induced HDAC4 inhibition was concordant with the earlier research that HBx can relieve a block on *CDKN1A* expression and prolong G1→S transition in human hepatoma cells [156,157]. Even though p21, the protein encoded by *CDKN1A*, is treated as a cyclin-dependent kinase inhibitor capable of inhibiting all cyclin/CDK complexes, it often promotes the assembly of type-D cyclins with CDK4 and CDK6 and arrests cell cycle progression in the G1 phase [156,158], which not only protects cells against apoptosis but also enhances the chance of tumorigenesis.

### 4.2. Autophagy in HBV Infection

Autophagy is a catabolic mechanism known to engulf long-lived proteins and damaged organelles via a lysosomal degradative pathway. Previous studies have suggested that HBV can hijack components in the autophagic regulatory pathways to promote its survival through augmentation of autophagosomes formation and inhibition of autolysosome maturation [159,160], which indicates the possibility of targeting the autophagic pathway for the treatment of Hepatitis B.

HBx-catalyzed autophagic response, which is crucial for the formation of autophagosomes as well as intensification of viral replication, resided mainly in the increased activity of the Beclin-1/Vps34 complex accompanied by the unchanged mTOR activity and positive regulation of *LC3* during HBV infection, which has also been reported in several research [159,160]. The phagophores undergo nucleation and elongation with Beclin-1/Vps34 complex and LC3 lipidation to form double-membraned autophagosomes [161], and the autophagic membranes generated during infection are used for viral envelopment [162]. However, rather than Beclin-1, only PI3KC3 was activated by the direct interconnection with HBx, which is in agreement with the study that the need for HBx to also induce the expression of Beclin-1 is redundant [163]. Moreover, although emerging study revealed that HBx efficiently impedes autophagic degradation by disturbing lysosomal acidification relevant to its degradative capacity without influencing on the fusion of autophagosomes and lysosomes [160], HBx-induced reduction of HDAC6 regulation ability might lead to autophagosome maturation failure [103]. Despite the necessity of further validation through biological experiments, this discovery provides possible mechanistic insight into the overall observation of HBV infection. Firstly, the intrinsic ability of HDAC6 to deacetylate NF-κB, having been detected to hamper the invasion of cancer [164,165], was destructed by HBx through transrepression, which might gradually create a potent microenvironment favorable to the malignant transformation of cells. Meanwhile, the negative-regulation of HDAC6 in human HCCs and consequential loss of its tumor suppression ability supported by previous research [166] apparently advocate the hypothesis.

### 4.3. Inflammation and Innate Immune Response in HBV Infection

Inflammation and innate immune response represent a highly coordinated process aimed at fighting infection or tissue injury. When cells are subject to foreign invasion, the inflammatory response can ensure successful resolution of the condition and the restoration of tissue homeostasis. However, inappropriately controlled natural defense mechanisms will eventually lead to the progression of chronic diseases [167]. In general, innate immunity counts for a great deal in governing infection right after contact with the pathogen to limit the spread of the virus [168]. However, the exploitation of immune and inflammatory signaling pathways enables viruses to subvert antiviral immunity and replicate in the hostile environment [169].

Of all the proteins involved in the activation and execution of inflammation response, NF-κB stands out as being crucial for this process in diverse metazoan organisms. During HBV infection, NF-κB-dependent transcription accelerated by HBx not only triggered the anti-apoptosis mechanism in favor of viral persistence as mentioned earlier but also augmented the inflammatory response leading to hepatitis and cell transformation in accordance with the indication of previous study [170]. Concurrently, emerging research has also uncovered that the dysregulated continual synthesis of IL-6, an identified target of NF-κB, plays a pathological effect on chronic inflammation and autoimmunity [167], which obliquely emphasizes the magnitude of the correlation between abnormally regulated NF-κB and excessive inflammation. Besides, regarding viral immunoregulation, even though it is contradictory that HBx on one hand sensitized cells to inflammatory stimuli, but on the other positive-regulated PD-L1 through transactivation of c-Jun and activation of STAT3, the findings are consistent with the idea that production of PD-L1 transcripts pertained positively to the intensity of liver inflammation to prohibit and thereby evade the host immune response [171,172]. 

Moreover, unlike other proteins in their family which typically share common characteristics and functions, STAT1 and STAT3 poles apart and the mutual functional antagonism of them in T-cell-induced inflammation has also been elucidated [173]. Despite both serving as the target of miR-122, *SOCS1* was regulated by miR-122 and thus transactivated by HBx in the identified mechanism during HBV infection, while *SOCS3* did not. Therefore, we suggest that the abrogation of STATs activity by HBx to attenuate the interferon-mediated suppression of infection depends on STAT1 instead of STAT3. Meanwhile, blockades of IL-6- and IFNs-stimulated immune signaling pathways by viral proteins were through STAT1 rather than STAT3, which also supports the idea from another perspective. In addition, activation of STAT3 mediated by HBx also bolstered its unshakeable status on maintaining viral persistence. Likewise, inactivation of STAT1 mediated by HBV proteins counteracted its effects on the provocation of the innate antiviral system. Such phenomena have also been revealed in the evidence of numerous studies [174]. It is worth noting that the reported capacity of HBx to block STAT1 nuclear import by means of affecting its methylation status was partially through the communication with CBP in the identified host/pathogen cross-talk. In such a way, that STAT1 lost its ability to block cell cycle progression and hinder hepatoma cell transformation in a course of prolonged inflammatory injury. This led to continuous destruction and regeneration of hepatocytes, increasing the chances of genetic alterations. 

### 4.4. Discovery of Potential Drug Combination

Based on the significant biomarkers including AKT1, NFKBIA, EIF4EBP1, HDAC6, STAT1, STAT3, and TP53, the combination of agreeable multi-target drugs, i.e., Sorafenib, Nutlin-3, and Tenofovir, was eventually recommended as a potential treatment for later clinical researches of the multi-drug therapy treating HBV-infected patients. Meanwhile, to gain deeper insight into the discovery of promising drugs, in silico profiling using deep learning-based DTI model was performed to predict interactions (dockings) between available drugs and the identified biomarkers. Additionally, deciding whether a drug is ready for clinical trials greatly pertains to preclinical studies that yield preliminary efficacy, toxicity, safety, etc., information. Therefore, to further narrow down the scope and improve the reliability of the predicted drugs, the general pharmacological specifications for drug design including regulation ability, toxicity, and sensitivity were additionally adopted to evaluate the efficacy. 

However, the entire process of moving a drug to clinical trials is actually a long way to go. Better preclinical preparation and assessment can be beneficial to the approval of a new medicine or drug combination. Considering the common reasons for withdrawal of an approved drug, e.g., hepatotoxicity and adverse events as well as the safety and tolerability assessment of a drug such as HLA (human leukocyte antigen) test, is conducive to better finding an agreeable option for drug combination. 

Repositioning drugs to treat with both common and rare disease is gradually becoming an attractive proposition. Taking advantage of drug repositioning avenues provides a faster discovery and translation of clinically relevant drug combinations. Furthermore, aided with the expediting development of computational technology and low-cost sequencing approaches, mounting publicly accessible data will certainly open up a broad spectrum of drug discovery and repositioning. Consequently, by systematically integrating more extensive data and information for both drugs and targets, a more appropriate drug combination out of the scope of original medical indication can be unveiled to bring new hope to HBV therapeutics.

## 5. Conclusions

The advent of the genomic eras has presented researchers with a myriad of high throughput biological data, which spark the interests in diverse biology applications. In this study, to investigate the pathogenetic mechanism under HBV infection, we constructed candidate host/pathogen interspecies genetic and epigenetic interaction network (HPI-GEN) by big data mining. Then, with the help of the two-side RNA-seq data, system identification strategies were applied to trim the false positives from the candidate HPI-GEN to obtain the real HPI-GEN. Thereafter, based on the extraction of PNP and the annotation of KEGG pathways, interspecies cross-talk signaling pathways are investigated from the real HPI-GEN for pathogenic mechanism under HBV infection to identify significant biomarkers. Moreover, in order to discover promising drugs for the identified drug targets, we trained a deep learning-based DTI model to predict possible drug-target interactions. Eventually, with the consideration of general drug design specifications including regulation ability, toxicity, and sensitivity, a combination of multi-target drugs as a potential multiple-molecule drug was selected to abate HBV infection. It is worth pointing out that although only pathogenesis for HBV infection was investigated in this work, our unique workflow can also be utilized on a wide variety of disease in view of systems biology, holding utility to aid in diversified drug discovery and repurposing process. Meanwhile, along with the development of NGS (Next-Generation Sequencing) technology, additionally integrating analysis from available genomics data into our pipeline can reinforce better downstream analysis and perform more exact biological interpretation. As more extensive genomic and pharmacological data being considered, the proposed pipeline could help reveal a more exhaustive host/pathogen offensive and defensive mechanism under HBV infection and facilitate the development of optimal therapy ensuring greater therapeutic outcome. 

## Figures and Tables

**Table 1:** The total number of nodes in candidate interspecies host/pathogen interspecies genetic and epigenetic interaction network (HPI-GEN) and identified HPI-GEN during Hepatitis B Virus (HBV) infection.

Node	Candidate HPI-GEN	Real HPI-GEN
LncRNA node	143	137
MiRNA node	922	922
TF node	2477	1325
Receptor node	2105	2105
Virus node	4	4
Protein node	13,465	12,258
Total node	19,116	16,751

**Table 2:** The total number of edges in candidate interspecies HPI-GEN and identified interspecies HPI-GEN during HBV infection.

Edge	Candidate HPI-GEN	Real HPI-GEN
LncRNA-LncRNA	0	0
LncRNA-miRNA	0	0
LncRNA-TF	156	9
LncRNA-Receptor	2	0
LncRNA-Protein	90	8
LncRNA-Virus	8	1
MiRNA-LncRNA	510	303
MiRNA-MiRNA	96	86
MiRNA-TF	42,913	7909
MiRNA-Receptor	26,888	9694
MiRNA-Protein	146,144	65,561
MiRNA-Virus	39	15
TF-LncRNA	231	157
TF-MiRNA	1439	944
TF-TF	33,426	8061
TF-Receptor	14,948	6500
TF-Protein	76,441	39,574
TF-Virus	54	38
Receptor-LncRNA	26	15
Receptor-MiRNA	143	102
Receptor-TF	2364	644
Receptor-Receptor	1490	693
Receptor-Protein	7755	4560
Virus-LncRNA	5	3
Virus-MiRNA	109	80
Virus-TF	509	77
Virus-Receptor	164	72
Virus-Protein	682	276
PPIs	4,039,657	906,428
Total edge	4,396,289	1,051,810

**Table 3:** The pathway enrichment analysis of proteins by applying the DAVID in core HPI-GEN during HBV infection.

Term	Numbers	*p*-Value
MicroRNAs in cancer	103	8.6 × 10^−18^
Pathways in cancer	108	8.7 × 10^−10^
FoxO signaling pathway	49	5.6 × 10^−9^
Viral carcinogenesis	63	6.3 × 10^−8^
Hepatitis B	47	7.3 × 10^−7^
Cell cycle	39	1.6 × 10^−5^
MAPK signaling pathway	64	6.2 × 10^−5^
TNF signaling pathway	33	1.3 × 10^−4^
Ras signaling pathway	55	6.2 × 10^−4^
PI3K-Akt signaling pathway	77	7.6 × 10^−4^

**Table 4:** The drug targets identified for HBV infection.

Disease	Drug Targets
HBV infection	AKT1, NFKBIA, EIF4EBP1, HDAC6, STAT1, STAT3, TP53

**Table 5:** Model performance (10-fold cross validation).

	Validation Loss	Validation Accuracy (%)	Testing Loss	Testing Accuracy (%)
**1**	0.254831	92.09	0.236721	92.28
**2**	0.237422	91.89	0.2264	92.04
**3**	0.228304	92.35	0.226482	92.49
**4**	0.227555	92.62	0.228102	92.6
**5**	0.223151	92.55	0.218528	92.74
**6**	0.223817	93.04	0.227157	92.77
**7**	0.239133	92.39	0.225822	92.58
**8**	0.235536	92.93	0.226848	92.8
**9**	0.22345	93.1	0.218475	93.11
**10**	0.235327	92.21	0.219082	92.64
**Average**	0.232853	92.517	0.225362	92.605
**Standard deviation**	0.00983	0.409527	0.005559	0.294099

**Table 6:** The candidate multi-target drugs for the identified biomarkers.

**AKT1**			
Drug	Regulation ability (L1000)	Toxicity (LD50, mol/kg)	Sensitivity (EC50, nM)
ribavirin	−1.5283	1.9876	−1.18823
tacrolimus	−0.5897	2.7541	−1.50335
sorafenib	−0.3125	2.7885	−0.43586
tenofovir	−0.6152	2.955	−0.63588
meloxicam	−0.2726	3.4619	−0.68734
**NFKBIA**			
Drug	Regulation ability (L1000)	Toxicity (LD50, mol/kg)	Sensitivity (EC50, nM)
chlortalidone	0.4056	1.8623	0.05198
busulfan	2.501	2.3207	−0.44631
rifaximin	0.681	2.6259	0.03563
sorafenib	3.5635	2.7885	−0.43586
meloxicam	3.2603	3.4619	−0.68734
**EIF4EBP1**			
Drug	Regulation ability (L1000)	Toxicity (LD50, mol/kg)	Sensitivity (EC50, nM)
chlortalidone	0.4153	1.8623	0.05198
busulfan	0.7491	2.3207	−0.44631
rifaximin	1.7372	2.6259	0.03563
sorafenib	0.4733	2.7885	−0.43586
dactinomycin	0.2923	4.3767	0.00307
**HDAC6**			
Drug	Regulation ability (L1000)	Toxicity (LD50, mol/kg)	Sensitivity (EC50, nM)
argatroban	0.5717	1.5976	−0.01405
chlortalidone	0.6495	1.8623	0.05198
entecavir	0.5388	2.3879	0.03615
nutlin-3	0.1749	2.529	−0.6695
meloxicam	0.1047	3.4619	−0.68734
**STAT1**			
Drug	Regulation ability (L1000)	Toxicity (LD50, mol/kg)	Sensitivity (EC50, nM)
chlortalidone	0.234	1.8623	0.05198
ribavirin	0.982	1.9876	−1.18823
zafirlukast	0.2615	2.5723	−0.24097
sorafenib	0.5799	2.7885	−0.43586
hydroflumethiazide	1.5238	3.1299	0.3949
**STAT3**			
Drug	Regulation ability (L1000)	Toxicity (LD50, mol/kg)	Sensitivity (EC50, nM)
argatroban	−1.2902	1.5976	−0.01405
ribavirin	−0.8934	1.9876	−1.18823
lamivudine	−0.3132	2.1348	−1.33248
zafirlukast	−0.5053	2.5723	−0.24097
tenofovir	−1.5088	2.955	−0.63588
hydroflumethiazide	−0.7348	3.1299	0.3949
meloxicam	−1.6727	3.4619	−0.68734
**TP53**			
Drug	Regulation ability (L1000)	Toxicity (LD50, mol/kg)	Sensitivity (EC50, nM)
acetylcysteine	1.0705	1.294	−1.2170
busulfan	2.2886	2.3207	−0.44631
nutlin-3	1.6295	2.529	−0.6695
sorafenib	0.5683	2.7885	−0.43586
calcitriol	0.4321	5.1352	0.28577

**Table 7:** Potential multiple-molecule drug and the corresponding target genes for HBV infection therapy.

Targets	AKT1	NFKBIA	EIF4EBP1	HDAC6	STAT1	STAT3	TP53
Drugs							
Sorafenib	O	O	O		O		O
Nutlin-3				O			O
Tenofovir	O					O	
Chemical structures of multi-target drugs							
Sorafenib			Nutlin-3		Tenofovir		
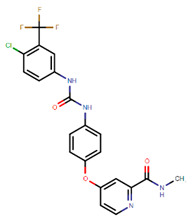			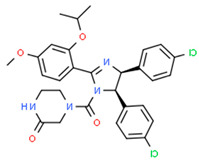		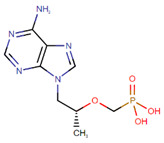		

O indicates the docking between each multi-target drug composing the multiple-molecule drug and their corresponding drug target.

## Appendix A

In order to avoid overfitting in the parameter estimation process, the cubic spline interpolation method was employed to interpolate 4 time points into 1440 points. Even if the cubic interpolation couldn’t increase the information for the parameter estimation, it could indeed prevent the parameters estimation in Equations (13) and (14) and (27)–(30) from overfitting during the identification process.

## References

[1] Chang M.-H. (2007). Hepatitis B virus infection. Semin. Fetal Neonatal Med..

[2] (2017). World Health Organization Global Hepatitis Report.

[3] Hong X., Kim E.S., Guo H. (2017). Epigenetic regulation of hepatitis B virus covalently closed circular DNA: Implications for epigenetic therapy against chronic hepatitis B. Hepatology.

[4] Fanning G.C., Zoulim F., Hou J., Bertoletti A. (2019). Therapeutic strategies for hepatitis B virus infection: Towards a cure. Nat. Rev. Drug Discov..

[5] Gehring A.J., Protzer U. (2019). Targeting innate and adaptive immune responses to cure chronic HBV Infection. Gastroenterology.

[6] Pol S., Nalpas B., Driss F., Michel M.-L., Tiollais P., Denis J., Bréchot C. (2001). Efficacy and limitations of a specific immunotherapy in chronic hepatitis B. J. Hepatol..

[7] Shih C., Chou S.-F., Yang C.-C., Huang J.-Y., Choijilsuren G., Jhou R.-S. (2016). Control and eradication strategies of hepatitis B virus. Trends Microbiol..

[8] Nassal M. (2015). HBV cccDNA: Viral persistence reservoir and key obstacle for a cure of chronic hepatitis B. Gut.

[9] Nosengo N. (2016). Can you teach old drugs new tricks?. Nature.

[10] Scannell J.W., Blanckley A., Boldon H., Warrington B. (2012). Diagnosing the decline in pharmaceutical R&D efficiency. Nat. Rev. Drug Discov..

[11] Rao V.S., Srinivas K., Sujini G.N., Kumar G.N.S. (2014). Protein-protein interaction detection: Methods and analysis. Int J. Proteom..

[12] Droit A., Poirier G., Hunter J. (2005). Experimental and bioinformatic approaches for interrogating protein-protein interactions to determine protein function. J. Mol. Endocrinol..

[13] Ashburn T.T., Thor K.B. (2004). Drug repositioning: Identifying and developing new uses for existing drugs. Nat. Rev. Drug Discov..

[14] Antolin A.A., Workman P., Mestres J., Al-Lazikani B. (2016). Polypharmacology in precision oncology: Current applications and future prospects. Curr. Pharm. Des..

[15] Mueller H., Wildum S., Luangsay S., Walther J., Lopez A., Tropberger P., Ottaviani G., Lu W., Parrott N., Zhang J.D. (2017). A novel orally available small molecule that inhibits hepatitis B virus expression. J. Hepatol..

[16] Stark C., Breitkreutz B.-J., Reguly T., Boucher L., Breitkreutz A., Tyers M. (2006). BioGRID: A general repository for interaction datasets. Nucleic Acids Res..

[17] Salwinski L., Miller C.S., Smith A.J., Pettit F.K., Bowie J.U., Eisenberg D. (2004). The database of interacting proteins: 2004 update. Nucleic Acids Res..

[18] Bader G.D., Betel D., Hogue C.W.V. (2003). BIND: The biomolecular interaction network database. Nucleic Acids Res..

[19] Orchard S., Ammari M., Aranda B., Breuza L., Briganti L., Broackes-Carter F., Campbell N.H., Chavali G., Chen C., del-Toro N. (2014). The MIntAct project--IntAct as a common curation platform for 11 molecular interaction databases. Nucleic Acids Res..

[20] Licata L., Briganti L., Peluso D., Perfetto L., Iannuccelli M., Galeota E., Sacco F., Palma A., Nardozza A.P., Santonico E. (2012). MINT, the molecular interaction database: 2012 update. Nucleic Acids Res..

[21] Min H., Yoon S. (2010). Got target? Computational methods for microRNA target prediction and their extension. Exp. Mol. Med..

[22] Friard O., Re A., Taverna D., De Bortoli M., Corá D. (2010). CircuitsDB: A database of mixed microRNA/transcription factor feed-forward regulatory circuits in human and mouse. BMC Bioinform..

[23] Zheng G., Tu K., Yang Q., Xiong Y., Wei C., Xie L., Zhu Y., Li Y. (2008). ITFP: An integrated platform of mammalian transcription factors. Bioinformatics.

[24] Bovolenta L.A., Acencio M.L., Lemke N. (2012). HTRIdb: An open-access database for experimentally verified human transcriptional regulation interactions. BMC Genom..

[25] Wingender E., Chen X., Hehl R., Karas H., Liebich I., Matys V., Meinhardt T., Prüss M., Reuter I., Schacherer F. (2000). TRANSFAC: An integrated system for gene expression regulation. Nucleic Acids Res..

[26] Calderone A., Licata L., Cesareni G. (2014). VirusMentha: A new resource for virus-host protein interactions. Nucleic Acids Res..

[27] Orchard S., Kerrien S., Abbani S., Aranda B., Bhate J., Bidwell S., Bridge A., Briganti L., Brinkman F.S.L., Cesareni G. (2012). Protein interaction data curation: The International Molecular Exchange (IMEx) consortium. Nat. Methods.

[28] del-Toro N., Dumousseau M., Orchard S., Jimenez R.C., Galeota E., Launay G., Goll J., Breuer K., Ono K., Salwinski L. (2013). A new reference implementation of the PSICQUIC web service. Nucleic Acids Res..

[29] Wu Z.-J., Zhu Y., Huang D.-R., Wang Z.-Q. (2010). Constructing the HBV-human protein interaction network to understand the relationship between HBV and hepatocellular carcinoma. J. Exp. Clin. Cancer Res..

[30] Qureshi A., Thakur N., Monga I., Thakur A., Kumar M. (2014). VIRmiRNA: A comprehensive resource for experimentally validated viral miRNAs and their targets. Database.

[31] Li C.-W., Wang W.-H., Chen B.-S. (2016). Investigating the specific core genetic-and-epigenetic networks of cellular mechanisms involved in human aging in peripheral blood mononuclear cells. Oncotarget.

[32] Xiao F., Zuo Z., Cai G., Kang S., Gao X., Li T. (2008). miRecords: An integrated resource for microRNA–target interactions. Nucleic Acids Res..

[33] Li J.-H., Liu S., Zhou H., Qu L.-H., Yang J.-H. (2013). starBase v2.0: Decoding miRNA-ceRNA, miRNA-ncRNA and protein–RNA interaction networks from large-scale CLIP-Seq data. Nucleic Acids Res..

[34] Hsu S.-D., Lin F.-M., Wu W.-Y., Liang C., Huang W.-C., Chan W.-L., Tsai W.-T., Chen G.-Z., Lee C.-J., Chiu C.-M. (2010). miRTarBase: A database curates experimentally validated microRNA–target interactions. Nucleic Acids Res..

[35] Kim D., Lee J., So C., Jeon H., Jeong M., Choi Y., Yoon W., Sung M., Kang J. (2019). A neural named entity recognition and multi-type normalization tool for biomedical text mining. IEEE Access.

[36] Lee J., Yoon W., Kim S., Kim D., Kim S., So C., Kang J. (2019). BioBERT: A pre-trained biomedical language representation model for biomedical text mining. Bioinformatics.

[37] Liu H., Hu Z.-Z., Zhang J., Wu C. (2005). BioThesaurus: A web-based thesaurus of protein and gene names. Bioinformatics.

[38] Vogel C., Marcotte E.M. (2012). Insights into the regulation of protein abundance from proteomic and transcriptomic analyses. Nat. Rev. Genet..

[39] Eulalio A., Huntzinger E., Izaurralde E. (2008). Getting to the root of miRNA-Mediated gene silencing. Cell.

[40] Zhang S., Pointer D., Singer G., Feng Y., Park K., Zhao L.J. (1998). Direct binding to nucleic acids by Vpr of human immunodeficiency virus type 1. Gene.

[41] Williams J.S., Andrisani O.M. (1995). The hepatitis B virus X protein targets the basic region-leucine zipper domain of CREB. Proc. Natl. Acad. Sci. USA.

[42] Cheong J.H., Yi M., Lin Y., Murakami S. (1995). Human RPB5, a subunit shared by eukaryotic nuclear RNA polymerases, binds human hepatitis B virus X protein and may play a role in X transactivation. EMBO J..

[43] Maguire H.F., Hoeffler J.P., Siddiqui A. (1991). HBV X protein alters the DNA binding specificity of CREB and ATF-2 by protein-protein interactions. Science.

[44] Kuzhandaivelu N., Cong Y.S., Inouye C., Yang W.M., Seto E. (1996). XAP2, a novel hepatitis B virus X-associated protein that inhibits X transactivation. Nucleic Acids Res..

[45] Ganem D., Prince A.M. (2004). Hepatitis B virus infection--natural history and clinical consequences. N. Engl. J. Med..

[46] Mitra B., Thapa R.J., Guo H., Block T.M. (2018). Host functions used by hepatitis B virus to complete its life cycle: Implications for developing host-targeting agents to treat chronic hepatitis B. Antivir. Res..

[47] Walsh D., Mohr I. (2011). Viral subversion of the host protein synthesis machinery. Nat. Rev. Microbiol..

[48] Lu P., Vogel C., Wang R., Yao X., Marcotte E.M. (2007). Absolute protein expression profiling estimates the relative contributions of transcriptional and translational regulation. Nat. Biotechnol..

[49] Anderson L., Seilhamer J. (1997). A comparison of selected mRNA and protein abundances in human liver. Electrophoresis.

[50] de Sousa Abreu R., Penalva L.O., Marcotte E.M., Vogel C. (2009). Global signatures of protein and mRNA expression levels. Mol. Biosyst..

[51] Maier T., Güell M., Serrano L. (2009). Correlation of mRNA and protein in complex biological samples. FEBS Lett..

[52] Wang Y.C., Chen B.S. (2010). Integrated cellular network of transcription regulations and protein-protein interactions. BMC Syst. Biol..

[53] The UniProt C. (2018). UniProt: A worldwide hub of protein knowledge. Nucleic Acids Res..

[54] Knox C., Law V., Jewison T., Liu P., Ly S., Frolkis A., Pon A., Banco K., Mak C., Neveu V. (2011). DrugBank 3.0: A comprehensive resource for ’omics’ research on drugs. Nucleic Acids Res..

[55] Gaulton A., Bellis L.J., Bento A.P., Chambers J., Davies M., Hersey A., Light Y., McGlinchey S., Michalovich D., Al-Lazikani B. (2012). ChEMBL: A large-scale bioactivity database for drug discovery. Nucleic Acids Res..

[56] Kim S., Thiessen P.A., Bolton E.E., Chen J., Fu G., Gindulyte A., Han L., He J., He S., Shoemaker B.A. (2016). PubChem Substance and Compound databases. Nucleic Acids Res..

[57] Liu T., Lin Y., Wen X., Jorissen R.N., Gilson M.K. (2006). BindingDB: A web-accessible database of experimentally determined protein–ligand binding affinities. Nucleic Acids Res..

[58] Khan S.A., Virtanen S., Kallioniemi O.P., Wennerberg K., Poso A., Kaski S. (2014). Identification of structural features in chemicals associated with cancer drug response: A systematic data-driven analysis. Bioinform. Oxf. Engl..

[59] Nandy A., Harle M., Basak S. (2006). Mathematical descriptors of DNA sequences: Development and applications. Gen. Pap. Ark..

[60] Gron A. (2017). Hands-On Machine Learning with Scikit-Learn. and TensorFlow: Concepts, Tools, and Techniques to Build. Intelligent Systems.

[61] Raschka S. (2015). Python Machine Learning.

[62] Abdi H., Williams L.J. (2010). Principal component analysis. WIREs Comput. Stat..

[63] Goodfellow I., Bengio Y., Courville A. (2016). Deep Learning.

[64] Kingma D., Ba J. (2014). Adam: A Method for Stochastic Optimization. arXiv.

[65] Bradley A.P. (1997). The use of the area under the ROC curve in the evaluation of machine learning algorithms. Pattern Recognit..

[66] Tiollais P., Pourcel C., Dejean A. (1985). The hepatitis B virus. Nature.

[67] Ganem D., Varmus H.E. (1987). THE molecular biology of the hepatitis B viruses. Annu. Rev. Biochem..

[68] Tuttleman J.S., Pourcel C., Summers J. (1986). Formation of the pool of covalently closed circular viral DNA in hepadnavirus-infected cells. Cell.

[69] Zakaria M.K., Sankhyan A., Ali A., Fatima K., Azhar E., Qadri I. (2014). HBV/HCV Infection and Inflammation. J. Genet. Syndr. Gene Ther..

[70] Chen L., Deng H., Cui H., Fang J., Zuo Z., Deng J., Li Y., Wang X., Zhao L. (2017). Inflammatory responses and inflammation-associated diseases in organs. Oncotarget.

[71] Garcia-Gomez A., Rodríguez-Ubreva J., Ballestar E. (2018). Epigenetic interplay between immune, stromal and cancer cells in the tumor microenvironment. Clin. Immunol..

[72] Ma Z., Cao Q., Xiong Y., Zhang E., Lu M. (2018). Interaction between hepatitis B virus and toll-like receptors: Current status and potential therapeutic use for chronic hepatitis B. Vaccines.

[73] Jacobs M.D., Harrison S.C. (1998). Structure of an IκBα/NF-κB Complex. Cell.

[74] Tanaka T., Narazaki M., Kishimoto T. (2014). IL-6 in inflammation, immunity, and disease. Cold Spring Harb. Perspect. Biol..

[75] Lee Y., Park U.S., Choi I., Yoon S.K., Park Y.M., Lee Y.I. (1998). Human interleukin 6 gene is activated by hepatitis B virus-X protein in human hepatoma cells. Clin. Cancer Res..

[76] Ivashkiv L.B., Donlin L.T. (2014). Regulation of type I interferon responses. Nat. Rev. Immunol..

[77] Schoenborn J., Wilson C. (2007). Regulation of Interferon-γ During Innate and Adaptive Immune Responses. Adv. Immunol..

[78] Nolan G.P., Fujita T., Bhatia K., Huppi C., Liou H.C., Scott M.L., Baltimore D. (1993). The bcl-3 proto-oncogene encodes a nuclear I kappa B-like molecule that preferentially interacts with NF-kappa B p50 and p52 in a phosphorylation-dependent manner. Mol. Cell. Biol..

[79] Kashatus D., Cogswell P., Baldwin A.S. (2006). Expression of the Bcl-3 proto-oncogene suppresses p53 activation. Genes Dev..

[80] Dann S.G., Thomas G. (2006). The amino acid sensitive TOR pathway from yeast to mammals. FEBS Lett..

[81] Lee D.-F., Kuo H.-P., Chen C.-T., Hsu J.-M., Chou C.-K., Wei Y., Sun H.-L., Li L.-Y., Ping B., Huang W.-C. (2007). IKKβ suppression of TSC1 Links inflammation and tumor angiogenesis via the Mtor pathway. Cell.

[82] Richter J.D., Sonenberg N. (2005). Regulation of cap-dependent translation by eIF4E inhibitory proteins. Nature.

[83] Mohr I. (2006). Phosphorylation and dephosphorylation events that regulate viral mRNA translation. Virus Res..

[84] Ryu D.-K., Ahn B.-Y., Ryu W.-S. (2010). Proximity between the cap and 5′ ε stem–loop structure is critical for the suppression of pgRNA translation by the hepatitis B viral polymerase. Virology.

[85] Sugatani T., Hruska K.A. (2005). Akt1/Akt2 and mammalian target of rapamycin/Bim play critical roles in osteoclast differentiation and survival, respectively, whereas Akt is dispensable for cell survival in isolated osteoclast precursors. J. Biol. Chem..

[86] Inoki K., Li Y., Zhu T., Wu J., Guan K.-L. (2002). TSC2 is phosphorylated and inhibited by Akt and suppresses mTOR signalling. Nat. Cell Biol..

[87] Ogawara Y., Kishishita S., Obata T., Isazawa Y., Suzuki T., Tanaka K., Masuyama N., Gotoh Y. (2002). Akt Enhances Mdm2-mediated Ubiquitination and Degradation of p53. J. Biol. Chem..

[88] Chiu A.P., Tschida B.R., Sham T.-T., Lo L.H., Moriarity B.S., Li X.-X., Lo R.C., Hinton D.E., Rowlands D.K., Chan C.-O. (2019). HBx-K130M/V131I promotes liver cancer in transgenic mice via AKT/FOXO1 signaling pathway and arachidonic acid metabolism. Mol. Cancer Res..

[89] Burton T.R., Gibson S.B. (2009). The role of Bcl-2 family member BNIP3 in cell death and disease: NIPping at the heels of cell death. Cell Death Differ..

[90] Liu J., Cao L., Chen J., Song S., Lee I.H., Quijano C., Liu H., Keyvanfar K., Chen H., Cao L.-Y. (2009). Bmi1 regulates mitochondrial function and the DNA damage response pathway. Nature.

[91] Alkema M., Wiegant J., Raap A.K., Bems A., van Lohuizen M. (1993). Characterization and chromosomal localization of the human proto-oncogene BMI-1. Hum. Mol. Genet..

[92] Danial N.N., Korsmeyer S.J. (2004). Cell Death: Critical Control Points. Cell.

[93] Oh H.-M., Yu C.-R., Dambuza I., Marrero B., Egwuagu C.E. (2012). STAT3 protein interacts with Class O Forkhead transcription factors in the cytoplasm and regulates nuclear/cytoplasmic localization of FoxO1 and FoxO3a proteins in CD4(+) T cells. J. Biol. Chem..

[94] Shlomai A., Shaul Y. (2009). The metabolic activator FOXO1 binds hepatitis B virus DNA and activates its transcription. Biochem. Biophys. Res. Commun..

[95] Hirata Y., Takahashi M., Morishita T., Noguchi T., Matsuzawa A. (2017). Post-Translational modifications of the TAK1-TAB complex. Int J. Mol. Sci..

[96] Shaulian E., Karin M. (2002). AP-1 as a regulator of cell life and death. Nat. Cell Biol..

[97] Schonthaler H.B., Guinea-Viniegra J., Wagner E.F. (2011). Targeting inflammation by modulating the Jun/AP-1 pathway. Ann. Rheum. Dis..

[98] Akira S. (2000). The role of IL-18 in innate immunity. Curr. Opin. Immunol..

[99] Qin W., Hu L., Zhang X., Jiang S., Li J., Zhang Z., Wang X. (2019). The diverse function of PD-1/PD-L pathway beyond cancer. Front. Immunol..

[100] Motokura T., Bloom T., Kim H.G., Jüppner H., Ruderman J.V., Kronenberg H.M., Arnold A. (1991). A novel cyclin encoded by a bcl1-linked candidate oncogene. Nature.

[101] Fujioka S., Niu J., Schmidt C., Sclabas G.M., Peng B., Uwagawa T., Li Z., Evans D.B., Abbruzzese J.L., Chiao P.J. (2004). NF-κB and AP-1 connection: Mechanism of NF-κB-Dependent regulation of AP-1 activity. Mol. Cell. Biol..

[102] Rebollo A., Dumoutier L., Renauld J.C., Zaballos A., Ayllón V., Martínez-A C. (2000). Bcl-3 expression promotes cell survival following interleukin-4 deprivation and is controlled by AP1 and AP1-like transcription factors. Mol. Cell. Biol..

[103] Lee J.-Y., Koga H., Kawaguchi Y., Tang W., Wong E., Gao Y.-S., Pandey U.B., Kaushik S., Tresse E., Lu J. (2010). HDAC6 controls autophagosome maturation essential for ubiquitin-selective quality-control autophagy. EMBO J..

[104] Aaronson D.S., Horvath C.M. (2002). A road map for those who don’t know JAK-STAT. Science.

[105] Stark G.R., Kerr I.M., Williams B.R., Silverman R.H., Schreiber R.D. (1998). How cells respond to interferons. Annu. Rev. Biochem..

[106] Ferrari S.M., Fallahi P., Ruffilli I., Elia G., Ragusa F., Paparo S.R., Patrizio A., Mazzi V., Colaci M., Giuggioli D. (2019). Immunomodulation of CXCL10 Secretion by hepatitis C Virus: Could CXCL10 be a prognostic marker of chronic hepatitis C?. J. Immunol. Res..

[107] Park J.-W., Li Z., Choi J.-S., Oh H.-J., Park S.-H., Yoon K.-C. (2012). Expression of CXCL9, -10, and -11 in the aqueous humor of patients with herpetic endotheliitis. Cornea.

[108] Nordmann A., Wixler L., Boergeling Y., Wixler V., Ludwig S. (2011). A new splice variant of the human guanylate-binding protein 3 mediates anti-influenza activity through inhibition of viral transcription and replication. FASEB J..

[109] Ueda T., Watanabe-Fukunaga R., Fukuyama H., Nagata S., Fukunaga R. (2004). Mnk2 and Mnk1 are essential for constitutive and inducible phosphorylation of eukaryotic initiation factor 4E but not for cell growth or development. Mol. Cell. Biol..

[110] Porter A.G., Jänicke R.U. (1999). Emerging roles of caspase-3 in apoptosis. Cell Death Differ..

[111] Stennicke H., Jürgensmeier J., Shin H., Deveraux Q., Wolf B., Yang X., Zhou Q., Ellerby H., Ellerby L., Bredesen D. (1998). Pro-caspase-3 Is a Major Physiologic Target of Caspase8. J. Biol. Chem..

[112] Wang J., Chun H.J., Wong W., Spencer D.M., Lenardo M.J. (2001). Caspase-10 is an initiator caspase in death receptor signaling. Proc. Natl. Acad. Sci. USA.

[113] Dikic I., Elazar Z. (2018). Mechanism and medical implications of mammalian autophagy. Nat. Rev. Mol. Cell Biol..

[114] Gottlieb T.M., Jackson S.P. (1993). The DNA-dependent protein kinase: Requirement for DNA ends and association with Ku antigen. Cell.

[115] Liu X., Zou H., Slaughter C., Wang X. (1997). DFF, a Heterodimeric Protein That Functions Downstream of Caspase-3 to Trigger DNA Fragmentation during Apoptosis. Cell.

[116] Dan H., Sun M., Kaneko S., Feldman R., Nicosia S., Wang H.-G., Tsang B., Cheng J. (2004). Akt Phosphorylation and Stabilization of X-linked Inhibitor of Apoptosis Protein (XIAP). J. Biol. Chem..

[117] Scott F.L., Denault J.-B., Riedl S.J., Shin H., Renatus M., Salvesen G.S. (2005). XIAP inhibits caspase-3 and -7 using two binding sites: Evolutionarily conserved mechanism of IAPs. EMBO J..

[118] Ahnesorg P., Smith P., Jackson S.P. (2006). XLF Interacts with the XRCC4-DNA Ligase IV Complex to Promote DNA Nonhomologous End-Joining. Cell.

[119] Wang S., Qiu L., Yan X., Jin W., Wang Y., Chen L., Wu E., Ye X., Gao G.F., Wang F. (2012). Loss of microRNA 122 expression in patients with hepatitis B enhances hepatitis B virus replication through cyclin G1-modulated P53 activity. Hepatology.

[120] Wang X.W., Gibson M.K., Vermeulen W., Yeh H., Forrester K., Stürzbecher H.W., Hoeijmakers J.H., Harris C.C. (1995). Abrogation of p53-induced apoptosis by the hepatitis B virus X gene. Cancer Res..

[121] Lee S.G., Rho H.M. (2000). Transcriptional repression of the human p53 gene by hepatitis B viral X protein. Oncogene.

[122] Moll U.M., Petrenko O. (2003). The MDM2-p53 Interaction. Mol. Cancer Res..

[123] Cregan S.P., MacLaurin J.G., Craig C.G., Robertson G.S., Nicholson D.W., Park D.S., Slack R.S. (1999). Bax-Dependent Caspase-3 Activation Is a Key Determinant in p53-Induced Apoptosis in Neurons. J. Neurosci..

[124] Chipuk J.E., Kuwana T., Bouchier-Hayes L., Droin N.M., Newmeyer D.D., Schuler M., Green D.R. (2004). Direct activation of bax by p53 mediates mitochondrial membrane permeabilization and apoptosis. Science.

[125] Sofer A., Lei K., Johannessen C.M., Ellisen L.W. (2005). Regulation of mTOR and cell growth in response to energy stress by REDD1. Mol. Cell. Biol..

[126] Ellisen L.W., Ramsayer K.D., Johannessen C.M., Yang A., Beppu H., Minda K., Oliner J.D., McKeon F., Haber D.A. (2002). REDD1, a developmentally regulated transcriptional target of p63 and p53, links p63 to regulation of reactive oxygen species. Mol. Cell.

[127] Pineau P., Volinia S., McJunkin K., Marchio A., Battiston C., Terris B., Mazzaferro V., Lowe S.W., Croce C.M., Dejean A. (2010). miR-221 overexpression contributes to liver tumorigenesis. Proc. Natl. Acad. Sci. USA.

[128] Fu X., Tan D., Hou Z., Hu Z., Liu G. (2012). miR-338-3p is down-regulated by hepatitis B virus X and inhibits cell proliferation by targeting the 3’-UTR region of CyclinD1. Int. J. Mol. Sci..

[129] Lu R., Yang Z., Xu G., Yu S. (2018). miR-338 modulates proliferation and autophagy by PI3K/AKT/mTOR signaling pathway in cervical cancer. Biomed. Pharmacother..

[130] Darnell J.E., Kerr I.M., Stark G.R. (1994). Jak-STAT pathways and transcriptional activation in response to IFNs and other extracellular signaling proteins. Science.

[131] Wu M., Xu Y., Lin S., Zhang X., Xiang L., Yuan Z. (2008). Hepatitis B virus polymerase inhibits the interferon-inducible MyD88 promoter by blocking nuclear translocation of Stat 1. J. Gen. Virol..

[132] Gao D., Zhai A., Qian J., Li A., Li Y., Song W., Zhao H., Yu X., Wu J., Zhang Q. (2015). Down-regulation of suppressor of cytokine signaling 3 by miR-122 enhances interferon-mediated suppression of hepatitis B virus. Antivir. Res..

[133] Li A., Song W., Qian J., Li Y., He J., Zhang Q., Li W., Zhai A., Kao W., Hu Y. (2013). MiR-122 modulates type I interferon expression through blocking suppressor of cytokine signaling 1. Int. J. Biochem. Cell Biol..

[134] Xavier G., Antoine B., Yoshua B. (2011). Deep sparse rectifier neural networks. PMLR.

[135] Srivastava N., Hinton G., Krizhevsky A., Sutskever I., Salakhutdinov R. (2014). Dropout: A simple way to prevent neural networks from overfitting. J. Mach. Learn. Res..

[136] Cao D.S., Zhang L.X., Tan G.S., Xiang Z., Zeng W.B., Xu Q.S., Chen A.F. (2014). Computational prediction of drug target interactions using chemical, biological, and network features. Mol. Inform..

[137] Byvatov E., Fechner U., Sadowski J., Schneider G. (2003). Comparison of support vector machine and artificial neural network systems for drug/nondrug classification. J. Chem. Inf. Comput. Sci..

[138] Liu Y., Wu M., Miao C., Zhao P., Li X.-L. (2016). Neighborhood regularized logistic matrix factorization for drug-target interaction prediction. PLoS Comput. Biol..

[139] LeBeau J.E. (1983). The role of the LD50 determination in drug safety evaluation. Regul. Toxicol. Pharmacol..

[140] Wilhelm S., Carter C., Lynch M., Lowinger T., Dumas J., Smith R.A., Schwartz B., Simantov R., Kelley S. (2006). Discovery and development of sorafenib: A multikinase inhibitor for treating cancer. Nat. Rev. Drug Discov..

[141] Ibrahim N., Yu Y., Walsh W.R., Yang J.L. (2012). Molecular targeted therapies for cancer: Sorafenib mono-therapy and its combination with other therapies (review). Oncol. Rep..

[142] Miao R., Xu X., Wang Z., Liu S., Qu K., Chen W., Liu C. (2018). Synergistic effect of nutlin-3 combined with aspirin in hepatocellular carcinoma HepG2 cells through activation of Bcl-2/Bax signaling pathway. Mol. Med. Rep..

[143] Tokalov S.V., Abolmaali N.D. (2010). Protection of p53 wild type cells from taxol by nutlin-3 in the combined lung cancer treatment. BMC Cancer.

[144] Zheng T., Wang J., Song X., Meng X., Pan S., Jiang H., Liu L. (2010). Nutlin-3 cooperates with doxorubicin to induce apoptosis of human hepatocellular carcinoma cells through p53 or p73 signaling pathways. J. Cancer Res. Clin. Oncol.

[145] Fernandez-Fernandez B., Montoya-Ferrer A., Sanz A.B., Sanchez-Niño M.D., Izquierdo M.C., Poveda J., Sainz-Prestel V., Ortiz-Martin N., Parra-Rodriguez A., Selgas R. (2011). Tenofovir nephrotoxicity: 2011 update. AIDS Res. Treat..

[146] Martin P., Lau D.T., Nguyen M.H., Janssen H.L., Dieterich D.T., Peters M.G., Jacobson I.M. (2015). A Treatment algorithm for the management of chronic hepatitis b virus infection in the united states: 2015 update. Clin. Gastroenterol. Hepatol..

[147] Lee Y.B., Lee J.-H., Lee D.H., Cho H., Ahn H., Choi W.-M., Cho Y.Y., Lee M., Yoo J.-J., Cho Y. (2014). Efficacy of entecavir-tenofovir combination therapy for chronic hepatitis b patients with multidrug-resistant strains. Antimicrob. Agents Chemother..

[148] Marcellin P., Ahn S.H., Ma X., Caruntu F.A., Tak W.Y., Elkashab M., Chuang W.-L., Lim S.-G., Tabak F., Mehta R. (2016). Combination of tenofovir disoproxil fumarate and peginterferon α-2a increases loss of hepatitis b surface antigen in patients with chronic hepatitis, B. Gastroenterology.

[149] Fung J., Lai C.L., Seto W.K., Yuen M.F. (2011). Nucleoside/nucleotide analogues in the treatment of chronic hepatitis B. J. Antimicrob. Chemother..

[150] Kew M.C. (2011). Hepatitis B virus x protein in the pathogenesis of hepatitis B virus-induced hepatocellular carcinoma. J. Gastroenterol. Hepatol..

[151] Diao J., Khine A.A., Sarangi F., Hsu E., Iorio C., Tibbles L.A., Woodgett J.R., Penninger J., Richardson C.D. (2001). X protein of hepatitis B virus inhibits Fas-mediated apoptosis and is associated with up-regulation of the SAPK/JNK pathway. J. Biol. Chem..

[152] Lee Y.I., Kang-Park S., Do S.I., Lee Y.I. (2001). The hepatitis B virus-X protein activates a phosphatidylinositol 3-kinase-dependent survival signaling cascade. J. Biol. Chem..

[153] Gottlob K., Fulco M., Levrero M., Graessmann A. (1999). The hepatitis B Virus HBx protein inhibits caspase 3 activity. J. Biol. Chem..

[154] Su F., Theodosis C.N., Schneider R.J. (2001). Role of NF-κB and myc proteins in apoptosis induced by hepatitis B virus HBx protein. J. Virol..

[155] Wang T., Wang Y., Wu M.-C., Guan X.-Y., Yin Z.-F. (2004). Activating mechanism of transcriptor NF-kappaB regulated by hepatitis B virus X protein in hepatocellular carcinoma. World J. Gastroenterol..

[156] Park U.S., Park S.K., Lee Y.I., Park J.G., Lee Y.I. (2000). Hepatitis B virus-X protein upregulates the expression of p21waf1/cip1 and prolongs G1-->S transition via a p53-independent pathway in human hepatoma cells. Oncogene.

[157] Al-Anazi M.R., Nazir N., Colak D., Al-Ahdal M.N., Al-Qahtani A.A. (2018). Deletion and functional analysis of hepatitis B virus X protein: Evidence for an effect on cell cycle regulators. Cell. Physiol. Biochem..

[158] Abbas T., Dutta A. (2009). p21 in cancer: Intricate networks and multiple activities. Nat. Rev. Cancer.

[159] Tang H., Da L., Mao Y., Li Y., Li D., Xu Z., Li F., Wang Y., Tiollais P., Li T. (2009). Hepatitis B virus X protein sensitizes cells to starvation-induced autophagy via up-regulation of beclin 1 expression. Hepatology.

[160] Liu B., Fang M., Hu Y., Huang B., Li N., Chang C., Huang R., Xu X., Yang Z., Chen Z. (2014). Hepatitis B virus X protein inhibits autophagic degradation by impairing lysosomal maturation. Autophagy.

[161] Rautou P.-E., Mansouri A., Lebrec D., Durand F., Valla D., Moreau R. (2010). Autophagy in liver diseases. J. Hepatol..

[162] Li J., Liu Y., Wang Z., Liu K., Wang Y., Liu J., Ding H., Yuan Z. (2011). Subversion of cellular autophagy machinery by hepatitis B virus for viral envelopment. J. Virol..

[163] Sir D., Tian Y., Chen W.-L., Ann D.K., Yen T.-S.B., Ou J.-H.J. (2010). The early autophagic pathway is activated by hepatitis B virus and required for viral DNA replication. Proc. Natl. Acad Sci. USA.

[164] Yang C.-J., Liu Y.-P., Dai H.-Y., Shiue Y.-L., Tsai C.-J., Huang M.-S., Yeh Y.-T. (2015). Nuclear HDAC6 inhibits invasion by suppressing NF-κB/MMP2 and is inversely correlated with metastasis of non-small cell lung cancer. Oncotarget.

[165] Kim M., Lu F., Zhang Y. (2016). Loss of HDAC-Mediated Repression and Gain of NF-κB Activation Underlie Cytokine Induction in ARID1A- and PIK3CA-Mutation-Driven Ovarian Cancer. Cell Rep..

[166] Jung K.H., Noh J.H., Kim J.K., Eun J.W., Bae H.J., Chang Y.G., Kim M.G., Park W.S., Lee J.Y., Lee S.Y. (2012). Histone deacetylase 6 functions as a tumor suppressor by activating c-Jun NH2-terminal kinase-mediated beclin 1-dependent autophagic cell death in liver cancer. Hepatology.

[167] Jones S.A. (2005). Directing transition from innate to acquired immunity: Defining a role for IL-6. J. Immunol..

[168] Busca A., Kumar A. (2014). Innate immune responses in hepatitis B virus (HBV) infection. Virol. J..

[169] Ong E.Z., Chan K.R., Ooi E.E. (2016). viral manipulation of host inhibitory receptor signaling for immune evasion. PLoS Pathog..

[170] Luedde T., Schwabe R.F. (2011). NF-κB in the liver--linking injury, fibrosis and hepatocellular carcinoma. Nat. Rev. Gastroenterol. Hepatol..

[171] Schönrich G., Raftery M.J. (2019). The PD-1/PD-L1 axis and virus infections: A delicate balance. Front. Cell Infect. Microbiol..

[172] Germanidis G., Argentou N., Hytiroglou P., Vassiliadis T., Patsiaoura K., Germenis A., Speletas M. (2013). Liver FOXP3 and PD1/PDL1 expression is down-regulated in chronic HBV hepatitis on maintained remission related to the degree of inflammation. Front. Immunol..

[173] Hong F., Jaruga B., Kim W.H., Radaeva S., El-Assal O.N., Tian Z., Nguyen V.-A., Gao B. (2002). Opposing roles of STAT1 and STAT3 in T cell–mediated hepatitis: Regulation by SOCS. J. Clin. Investig..

[174] Dufour J.-F., Clavien P.A. (2010). Signaling Pathways in Liver Diseases.

